# Comprehensive analysis of metabolic patterns in renal cell carcinoma: implications for prognosis and treatment

**DOI:** 10.3389/fimmu.2025.1630053

**Published:** 2025-09-17

**Authors:** Yue Wang, Pengfei Li, Tao Feng, Yonghao Chen, Wei Liu, Qintao Ge, Qingchuan Zhang

**Affiliations:** ^1^ Department of Urology, Putuo Hospital, Shanghai University of Traditional Chinese Medicine, Shanghai, China; ^2^ Department of Urology, The First Affiliated Hospital of Anhui Medical University, Hefei, Anhui, China; ^3^ Department of Urology, Fudan University Shanghai Cancer Center, Shanghai, China; ^4^ West China Medical Center of Sichuan University, Chengdu, Sichuan, China

**Keywords:** renal cell carcinoma, metabolic subtyping, prostaglandin biosynthesis, immuneexhaustion, tumor prognosis

## Abstract

**Introduction:**

Renal cell carcinoma (RCC) presents significant clinical and molecular heterogeneity, which makes prognosis and treatments very complicated. Despite advances in surgical and systemic therapies, a substantial number of RCC patients progress to advanced stages, highlighting the need for novel stratification approaches that account for the tumor’s biological complexity.

**Methods:**

An integrative multi-omic analysis, combining transcriptomic and clinical data, was performed to identify the metabolic subtypes of RCC. Unsupervised clustering was used to stratify patients based on their metabolic profiles, and subtype-specific molecular signatures were examined through differential expression and pathway enrichment analyses. Prognostic outcomes, immune features, and drug sensitivities were then analyzed. The value of the classification was validated by the biological experiments.

**Results:**

Three distinct metabolic subtypes (C1, C2, and C3) were identified, each associated with distinct survival outcomes. The C1 subtype, marked by enhanced oxidative phosphorylation and fatty acid metabolism, correlated with improved survival. The C2 subtype, characterized by prostaglandin biosynthesis, was linked to poor prognosis and immune evasion. The C3 subtype was similar to C2 but was characterized by extensive prostanoid biosynthesis, indicating a moderate prognosis in the three subtypes. Immunotherapy and targeted drug sensitivity analyses revealed subtype-specific vulnerabilities, suggesting potential therapeutic strategies tailored to each metabolic profile. Subsequent *in vitro* assays confirmed the significance of targets to the RCC biological process.

**Conclusions:**

Metabolic subtyping through multi-omics integration offers a clinically relevant framework for RCC prognosis and personalized treatment. This approach highlights the role of metabolic reprogramming in tumor immunity and therapeutic response, providing a foundation for future clinical applications in precision oncology.

## Introduction

Renal cell carcinoma (RCC) is one of the most common urological malignancies worldwide, ranking second only to bladder cancer in incidence (1). According to the authoritative statistics, more than 430,000 new cases of RCC and over 150,000 related deaths were reported in 2022 (2). Over the past few years, the incidence of RCC appeared to increase by approximately 1.5% per year (3), although the surgical treatments (radical resection or partial nephrectomy) represent the primary interventions and confirm positive outcomes. Despite the rapid technical improvement (robotic-assisted surgery and ablation treatment) addressing several of the limitations of traditional approaches, approximately 30% of RCC patients are still diagnosed at advanced stages (4); therefore, the necessity for multidisciplinary treatment (MDT) strategies has been increasingly emphasized.

Despite the growing diagnosis and multiple stages/grades, the incidence and mortality of RCC vary considerably. Data from GLOBOCAN 2020 indicate that men are twice as likely as women to develop and die from RCC (5). Meanwhile, RCC has strong regional and racial disparities, and it is thought to be driven by a combination of genomic alterations and lifestyle-associated risk factors (6, 7), contributing to the pronounced heterogeneity observed among RCC patients. Furthermore, based on the variety of molecular mechanisms, diverse classical research has discovered significant targets, signaling pathways, and corresponding pharmaceuticals, which aim at immunotherapy and targeted therapy in the advanced RCC patients (8, 9). Nevertheless, a significant proportion of patients remain refractory or are only partially responsive to current treatments. Combination therapies have therefore become necessary to overcome resistance, underscoring the urgent need to further dissect the complex molecular and metabolic landscapes of RCC.

The substantial heterogeneity of RCC encompasses diverse genetic, epigenetic, and metabolic alterations across histological subtypes. Recently, various kinds of sequencing methods have markedly advanced, and many novel techniques not only broaden the range of the internal pathways but also ensure that the potential mechanisms are closer to the actual conditions of RCC (10, 11). There have been more than 20 biomarkers found in RCC, which are specific to the mutant site in the microenvironment of RCC. The most frequent gene mutation is Von Hippel–Lindau (VHL), which directly dysregulates hypoxia-inducible factor (HIF) signaling and contributes to aberrant hypoxic responses within the tumor microenvironment (12). In addition, PBRM1 was the second most frequent mutation found in RCC, resulting in the break of the cellular chromatin-remodeling complex SWI/SNF, destroying DNA replication and cell proliferation (13). Moreover, KDM5C, BAP1, and SETD2 were identified to have certainly mutated in RCC (14, 15); also, some other targets are emerging along with the progress of sequencing. Molecular classification based on the above mutations may trigger the new generation of targeted therapies (16). Except for the molecules, PI3K, mTOR, VEGF, and other signaling pathways were recognized, and corresponding therapy, like tyrosine kinase inhibitor (TKI), has been used clinically (17). The outcomes of RCC patients are indeed better, but emerging data suggest an increasing incidence of therapy resistance, particularly among patients with metastatic RCC (mRCC). In response, first-line treatment strategies have evolved toward combination regimens involving two or more targeted agents or immunotherapies, as reflected in current clinical guidelines (18–20). These developments highlight the limitations of conventional molecular classifications and the necessity for novel stratification frameworks for RCC.

As a classical and powerful theory, the Warburg effect provides a more comprehensive perspective for cancer research, and aberrant cancer metabolism is regarded as the hallmark (21). RCC is characterized by profound metabolic disturbances throughout its initiation and progression (22), and “metabolic disorder” tends to be the essential feature of RCC. VHL–HIF targets and pathways are considered the fundamental programs of RCC. Abnormal glycolysis would be initiated, and energy metabolism dysfunction would appear in the tumor microenvironment. Instead, cancer cell proliferation and metastasis are uncontrolled while adjusting to the variable metabolic conditions (23). With the emergence of “the era of omics”, multiple critical metabolism patterns were unveiled in RCC. The pentose phosphate pathway is a crucial process *in vivo*, and the reprogramming is found to be correlated with the aggression in RCC (24). Otherwise, lipid metabolism, represented by the *de novo* fatty acid (FA)-related pathway, has been confirmed to be required for RCC, targeting associated biomarkers, like fatty acid synthase (FASN), stearoyl-CoA desaturase 1 (SCD1), and carnitine palmitoyltransferase 1A (CPT1A), which have been proposed to be potential clinical strategies (25). Furthermore, glutamine-derived and oncometabolite production pathways were discovered to be apparently unstable in RCC, the tricarboxylic acid cycle (TCA) was impeded, and the growth of cancer cells was accelerated (26). Hence, the metabolic characteristics of RCC call for a preferred classification to identify some specific targets.

In this study, we comprehensively explored the global metabolic patterns of RCC through multiple clustering and enrichment analyses. We attempted to demonstrate the relationship between clinical characteristics and metabolism and find the key pathways driving RCC progression. We validated metabolic patterns via relevant experiments. This new metabolism-associated classification may provide a new insight into the mechanisms and underlying therapies of RCC.

## Methods

### Data acquisition and processing

Transcriptomic and clinical data were integrated from three independent cohorts of clear cell renal cell carcinoma (ccRCC): TCGA-KIRC, EMTAB3267, and GSE22541. All the clinical annotations were carried out according to the platforms.

All datasets were filtered to include only primary tumor samples with complete survival data, tumor grades, and TNM staging. Raw data of RNA-seq were converted to transcripts per million (TPM) and transformed to log2(TPM+1). Microarray data were normalized using quantile normalization, and batch effects were removed using ComBat (**Supplementary Figure S1A**).

### Unsupervised clustering of metabolic subtypes

To identify distinct metabolic subtypes, unsupervised consensus clustering was performed on the TCGA-KIRC cohort using the ConsensusClusterPlus package (R v4.2.3) (27). Subsequently, principal component analysis (PCA) was applied to reduce the dimensionality of the original expression matrix, and the samples were projected into a two-dimensional space to intuitively display the separation of each metabolic subtype (Data sheet 1). K-means clustering with a Euclidean distance metric was employed, and the robustness of clustering was assessed across 1,000 iterations, each involving random subsampling of 80% of the samples. The optimal number of clusters was determined by integrating results from cumulative distribution function (CDF) plots, delta area analyses, and silhouette width metrics, thereby ensuring stable and well-defined subtype classification.

### Template-based molecular subtyping using nearest template prediction

To better stratify patients into biologically and clinically relevant subtypes, the nearest template prediction (NTP) approach in the “MOVICS” package was implemented (28) based on predefined molecular templates. Subtype-specific templates were constructed based on the average gene expression profiles of typical samples. Gene symbols were harmonized according to HUGO Gene Nomenclature Committee (HGNC) standards, and only the common genes in all datasets were retained for subsequent analyses.

### Gene set enrichment analysis for GO and KEGG pathways

Gene Ontology (GO) and Kyoto Encyclopedia of Genes and Genomes (KEGG) enrichment analyses were conducted using the clusterProfiler R package (version 4.6.2). Enrichment analysis was applied separately for biological processes (BPs), cellular components (CCs), and molecular functions (MFs) in GO terms and for metabolic and signaling pathways in KEGG. Pathways with an adjusted *p*-value <0.05 were considered significantly enriched.

### Microenvironment Cell Populations-counter

To estimate the absolute abundance of distinct stromal and immune cell populations from bulk transcriptomic profiles, we employed Microenvironment Cell Populations-counter (MCP-counter) by the “MCPcounter” R package (version 1.2.0, Bioconductor version 3.20) (29). This approach quantified specific cell populations based on cell type-specific transcriptomic markers. The immune cell populations assessed included the following: CD8+ T cells, total T cells, natural killer (NK) cells, cytotoxic lymphocytes, myeloid dendritic cells, monocytes, and neutrophils. Additionally, we estimated the abundance of stromal components, namely, endothelial cells and fibroblasts, for each sample. Furthermore, we performed six complementary algorithms—MCP-counter, CIBERSORTx, EPIC, quanTIseq, TIMER2.0, and xCell—based on the IOBR package (30) to decipher the immune infiltration pattern among high and low PTGES2 tumors.

### Single-sample gene set enrichment analysis

To further delineate heterogeneity in immune infiltration between samples, single-sample gene set enrichment analysis (ssGSEA) was performed using the “GSVA” R package (Bioconductor version 3.20) (31). An enrichment score for each gene set was computed, enabling a quantitative assessment of immune-related gene set activity at single-sample resolution by ranking the genes and comparing the distributions.

### GSVA-based characterization of immune cell and functional pathway activities

Gene set variation analysis (GSVA) was applied to RNA-seq expression profiles to estimate pathway activities and immune cell infiltration in an unsupervised, non-parametric manner. GSVA and enrichment scores were performed using the “GSVA” R package. Hierarchical clustering of GSVA scores enabled the visualization and identification of subtype-specific patterns in the tumor immune microenvironment (TIME), which are displayed graphically in the heatmaps. To evaluate the features of Tertiary lymphoid structure (TLS), Germinal centers B cell (GC B), Follicular helper T cell (Tfh), Follicular dendritic cells (FDC), Plasma, and B cells, GSVA was performed based on related gene sets, which were obtained from a previous study (32). In addition, immune-related signaling between high and low PTGES2 tumors enrolled from the Hallmark database was compared based on GSVA algorithms.

### Assessment of immunotherapy response in the CheckMate immunotherapy cohort

mRCC patients from the CheckMate immunotherapy cohort (16) who received immunotherapies were analyzed. Clinical response was categorized as follows: clinical benefit (CB), defined as complete response (CR), partial response (PR), or stable disease (SD) lasting ≥6 months; and no clinical benefit (NCB), defined as progressive disease (PD) or SD lasting <6 months. Objective response rates (ORRs), including CR, PR, SD, PD, and confirmed mixed partial response (CMPR), were calculated for every subtype.

### Drug sensitivity profiling of RCC

To investigate the differential drug sensitivity of RCC, the OncoPredict algorithms implemented in MOVICS were utilized alongside drug response data from the Genomics of Drug Sensitivity in Cancer (GDSC) database (Release 8.5, October 2023). The sensitivity of a range of therapeutic drugs was analyzed, and the half-maximal inhibitory concentration (IC_50_) values for each drug were estimated within the context of three distinct subtypes.

### Immunohistochemistry

Paraffin-embedded RCC tissues were deparaffinized in xylene and rehydrated through graded ethanol. Antigen retrieval was performed by boiling the sections in Tris-EDTA buffer (pH 9.0) for 15 minutes in an electric cooker, and then the sections were naturally cooled. Endogenous peroxidase activity was quenched using 3% hydrogen peroxide for 10 minutes at room temperature. Next, tissue sections were blocked with 3% bovine serum albumin (BSA) for 30 minutes and then incubated with primary antibodies (anti-PTGES2; dilution 1:200, Proteintech, Wuhan, Hubei, P.R.C, Cat No. 10881-1-AP) overnight at 4°C. The next day, sections were washed three times and incubated with Horseradish Peroxidase (HRP)-conjugated secondary antibodies (Absin Bioscience, China, No. abs996, general concentration) for 30 minutes at room temperature. Detection was performed using Diaminobenzidine (DAB) substrate, and counterstaining was performed using hematoxylin. Images were acquired using a Leica microscope and imaging system.

### Cell culture

Human RCC cell lines 786-O and 769-P were obtained from the American Type Culture Collection (ATCC, Mansas, Virginia, USA). To confirm the identity of the cell lines, short tandem repeat (STR) analysis was used to identify the two cell lines. STR testing was conducted by Shanghai Zhong Qiao Xin Zhou Biotechnology Co., Ltd. (**Supplementary Materials 2**, **3**). Cells were cultured in RPMI-1640 medium (Gibco, Thermo Fisher Scientific, Shanghai, China, A4192301) supplemented with 10% fetal bovine serum (FBS; Gibco, A5256701) and 1% penicillin–streptomycin (100 U/mL and 100 μg/mL, respectively). All cells were maintained in a humidified incubator at 37 °C with a 5% CO_2_ atmosphere and were routinely tested to be free of mycoplasma contamination. Cell passage occurred when cells grew to a density of 70%–80%. Notably, cells within 20 passages were fit for all experiments to ensure phenotypic stability.

### Construction of stable cell lines of PTGES2 knockdown and overexpression

To generate stable knockdown RCC cell lines, two short hairpin RNA (shRNA) sequences targeting human PTGES2 were designed, and the sequences were as follows: shPTGES2-1: 5′-GAAGCCGAATCTCGCTGATTT-3′, shPTGES2-2: 5′-CGGCAATAAGTACTGGCTCAT-3′. A scrambled non-targeting RNA (5′-CAACAAGATGAAGAGCACCAA-3′) was used as a negative control (shCtrl). All oligonucleotides were cloned into the pLKO.1-puro vector to generate two knockdown plasmids, and PTGES2 [Coding sequence (CDS) region was obtained from https://www.ncbi.nlm.nih.gov/nuccore/NM_025072.7] was cloned into a pCDH vector to generate an overexpression plasmid (33).

Lentiviral particles were produced by co-transfecting HEK293T cells with the plasmids and packaging plasmids (psPAX2 and pMD2.G) using Lipofectamine 3000 (Thermo Fisher Scientific). Viral supernatants were collected at 48 and 72 hours post-transfection.

For transduction, cells were seeded in six-well plates (2 × 10^5^ cells/well) and infected with lentivirus at a suitable multiplicity of infection (MOI; 30–50) value in the presence of 8 μg/mL polybrene (Sigma-Aldrich, Beijing, China). After 24 hours, the medium was replaced with fresh complete medium containing 2 μg/mL puromycin for 7 days to select stably transduced cells.

### Quantitative polymerase chain reaction

Total mRNA of cells was extracted using TRIzol Reagent (Invitrogen, Shanghai, China), and then mRNA was reverse-transcribed into cDNA using PrimeScript RT Master Mix (Takara Bio, Beijing, China) and quantified by qRT-PCR on a QuantStudio 7 Flex System. The following primers were used:

PTGES2: Forward: 5′-GTGACCGAGTTCGGCAATAAG-3′,
PTGES2: Reverse: 5′-CGGACAATGTAGTCAAAGGACG-3′,
GAPDH: Forward: 5′-GGAGCGAGATCCCTCCAAAAT-3′,
GAPDH: Reverse: 5′-GGCTGTTGTCATACTTCTCATGG-3′.

The run method is shown below:

Hold stage: 50°C for 2min, 95°C for 10 min.
PCR stage: 95°C for 15 s, 60°C for 1min and 40 cycles.
Melt curve stage: 95°C for 15 s, 60°C for 1min, 95°C for 15 s.

The relative mRNA expression is calculated using the following method:

ΔCt = Ct (PTGES2) − Ct (GAPDH),
ΔΔCt = ΔCt (Sample) − ΔCt (Control),
Fold gene expression = 2^−(ΔΔCt).

### Western blotting

Cells were lysed in Radio Immunoprecipitation Assay Lysis buffer (RIPA) supplemented with protease and phosphatase inhibitors (Beyotime, Shanghai, China) on ice for 30 minutes. Lysates were then centrifuged at 13,000 *g* for 15 minutes at 4°C, and protein concentrations were quantified using the BCA assay kit (Thermo Fisher Scientific). Equal amounts of protein (20–30 μg) were separated using the Sodium dodecyl sulfate - polyacrylamide gel electrophoresis (SDS-PAGE) gel (10%) and were transferred to Polyvinylidene Fluoride (PVDF) membranes (Millipore, Shanghai, China; 0.45 μm). Membranes were blocked in 5% non-fat milk for 1 hour at room temperature and incubated overnight at 4°C with primary antibodies (anti-PTGES2, dilution 1:1,000, Proteintech, Cat No. 10881-1-AP; loading control, beta actin, dilution 1:5,000, Proteintech, Cat No. 20536-1-AP).

The next day, membranes were washed three times and then incubated with HRP-conjugated secondary antibodies (HRP-conjugated Goat Anti-Rabbit IgG(H+L), dilution 1:5000, Proteintech, Cat No. SA00001-2) for 1 hour at room temperature. Signals were detected using enhanced chemiluminescence (ECL) reagents and visualized using an imaging system (Tanon chemiluminescence image analysis system).

### Cell proliferation assay

Cell viability was assessed using the Cell Counting Kit-8 reagent (Dojindo, Kumamoto, Japan) according to the manufacturer’s instructions. 786-O and 769-P cells were seeded into 96-well plates at a density of 3 × 10^3^ cells/well. After the cell adhesion was completed, 10 μL of CCK-8 reagent was added to each well and incubated for 2 hours at 37°C. Absorbance at OD 450 nm was measured. It was noteworthy that the cells should be measured at a fixed time every day and five times in total.

### Transwell assay

The Transwell assay was used to test the cell migration capabilities; 2 × 10^4^ cells suspended in serum-free medium were seeded into the upper Transwell chamber (8-μm pore size; Corning, Shanghai, China) in 24-well plates. The lower chamber contained 800 μL of complete medium with 10% FBS. After incubating for 24 hours at 37°C, cells on the upper membrane surface were gently removed using a cotton swab. The migrated cells on the bottom surface were fixed with 4% paraformaldehyde and then stained with 0.1% crystal violet for 30 minutes. Cell counting was carried out using an inverted microscope (Olympus, Beijing, China) in three random fields per well.

### Colony formation assay

To assess cell proliferative capacity, 786-O and 769-P cells were seeded into six-well plates at a low density of 1,000 cells/well. After culturing for 10–14 days, colonies were fixed with 4% paraformaldehyde for 30 minutes and stained with 0.1% crystal violet for 30 minutes. Visible colonies were counted.

### Statistical analysis

The Kaplan–Meier survival curves of defined metabolic subtypes were generated using the survival and survminer R packages. Differences in overall survival (OS) among subtypes were evaluated using the log-rank test. Multivariate Cox proportional hazards models were adjusted for age, gender, grade, TNM stage, and metastasis. The HRs and 95% CIs were calculated, and the proportional hazards assumption was strictly tested using Schoenfeld residuals. Correlations between clinical and molecular features were tested using χ^2^ or Fisher’s exact test (grade, stage, and metastasis) for categorical variables, and the Kruskal–Wallis or analysis of variance (ANOVA) (age and tumor size) was utilized for continuous variables.

The R software (v4.2.3) and GraphPad Prism (v9.0) were used for all statistical analyses. *p*-Values <0.05 were considered statistically significant.

## Results

### Three distinct global metabolic patterns presented different clinical features in ccRCC

**Figure 1:**
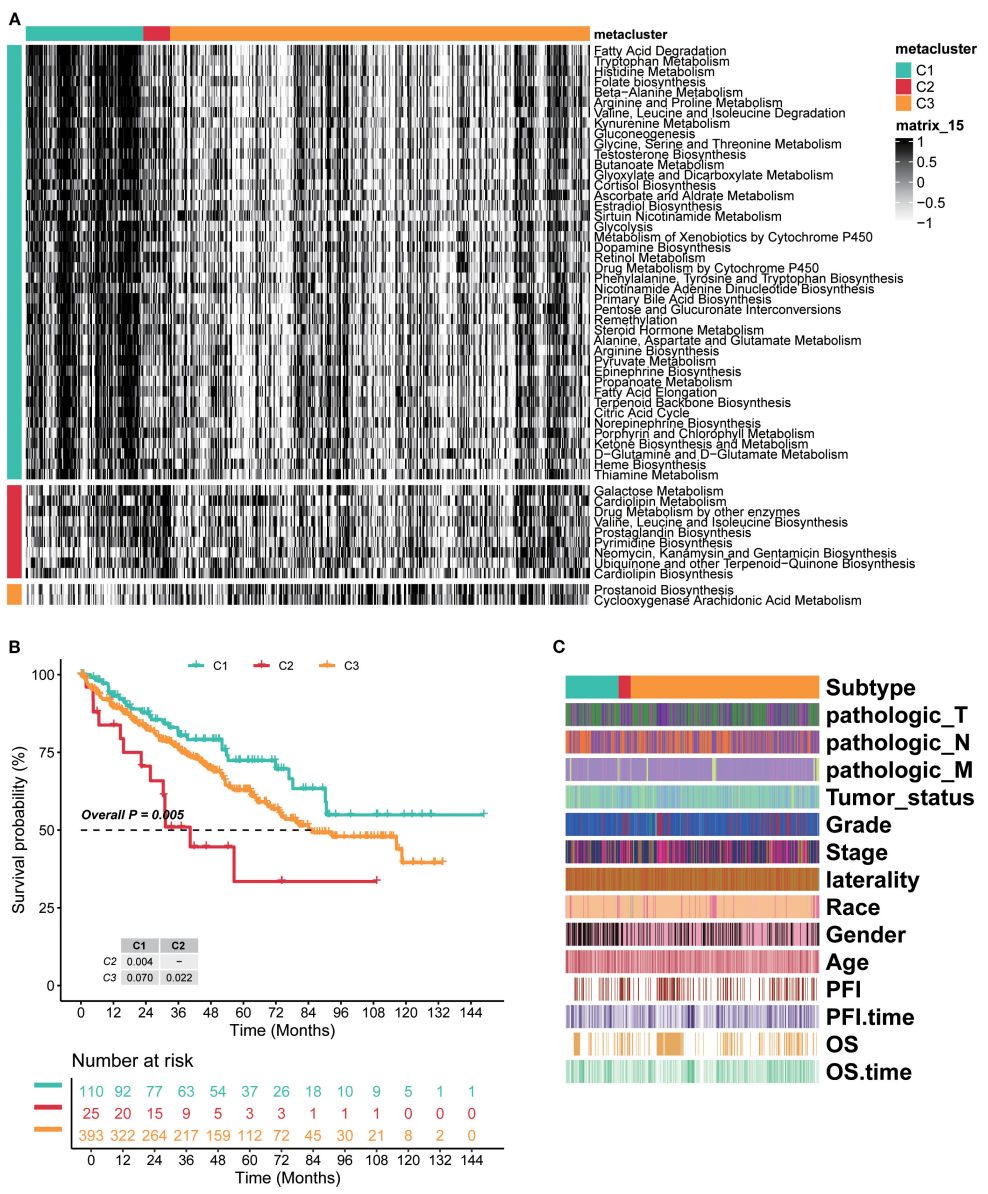
Three distinct global metabolic patterns presented different clinical features in ccRCC. **(A)** Unsupervised clustering of intersecting genes from TCGA-KIRC, EMTAB3267, and GSE22541 identified three metabolic subtypes. KEGG enrichment analysis indicated several significant metabolic pathways of three subtypes. **(B)** Kaplan–Meier curves of survival probability across three subtypes. **(C)** Heatmap of detailed clinical characteristics of patients across three subtypes. KEGG, Kyoto Encyclopedia of Genes and Genomes.

By integrating intersecting metadata of RCC samples from TCGA-KIRC, EMTAB3267, and GSE22541, three metabolic subtypes (C1, C2, and C3) were divided by unsupervised clustering (**Supplementary Figure S1B**). KEGG enrichment analysis indicated several significant metabolic pathways, as shown in **Figure 1A**. Genes in cluster C1 were mainly enriched in fatty acid degradation and metabolism of various amino acids (arginine, glycine, serine, etc.). Cluster C2 comprised galactose metabolism, cardiolipin metabolism, prostaglandin biosynthesis, and other metabolite biosynthesis processes. Cluster C3 included prostanoid biosynthesis and cyclooxygenase, arachidonic acid metabolism. These findings implicated metabolic rewiring as a key differentiator of ccRCC clinical behavior.

The Kaplan–Meier analysis showed marked survival differences among the three subtypes. Generally, cluster C1 had the better survival probability, C3 was in the middle, and C2 presented the worst prognosis (overall *p*=0.005, C1 vs. C2 *p*=0.004, C2 vs. C3 *p*=0.022, and C1 vs. C3 *p*=0.070; **Figure 1B**). The detailed clinical characteristics of patients were assessed under established clusters C1–C3. The results showed that pathologic_T, pathologic_N, and pathologic_M were strongly associated with every subtype, and these three almost entirely emerged in cluster C2, but presented a low proportion in clusters C1 and C3. Meanwhile, Progression Free Interval (PFI) and OS showed a similar tendency (**Figure 1C**). Therefore, the novel three clusters based on metabolism were distinctly classified for RCC and had strongly prognostic effects on patients. Overall, these results confirmed the reproducibility, prognostic utility, and biological validity of the metabolic subtype model.

### Validation of independent datasets revealed diverse outcomes of RCC patients

**Figure 2:**
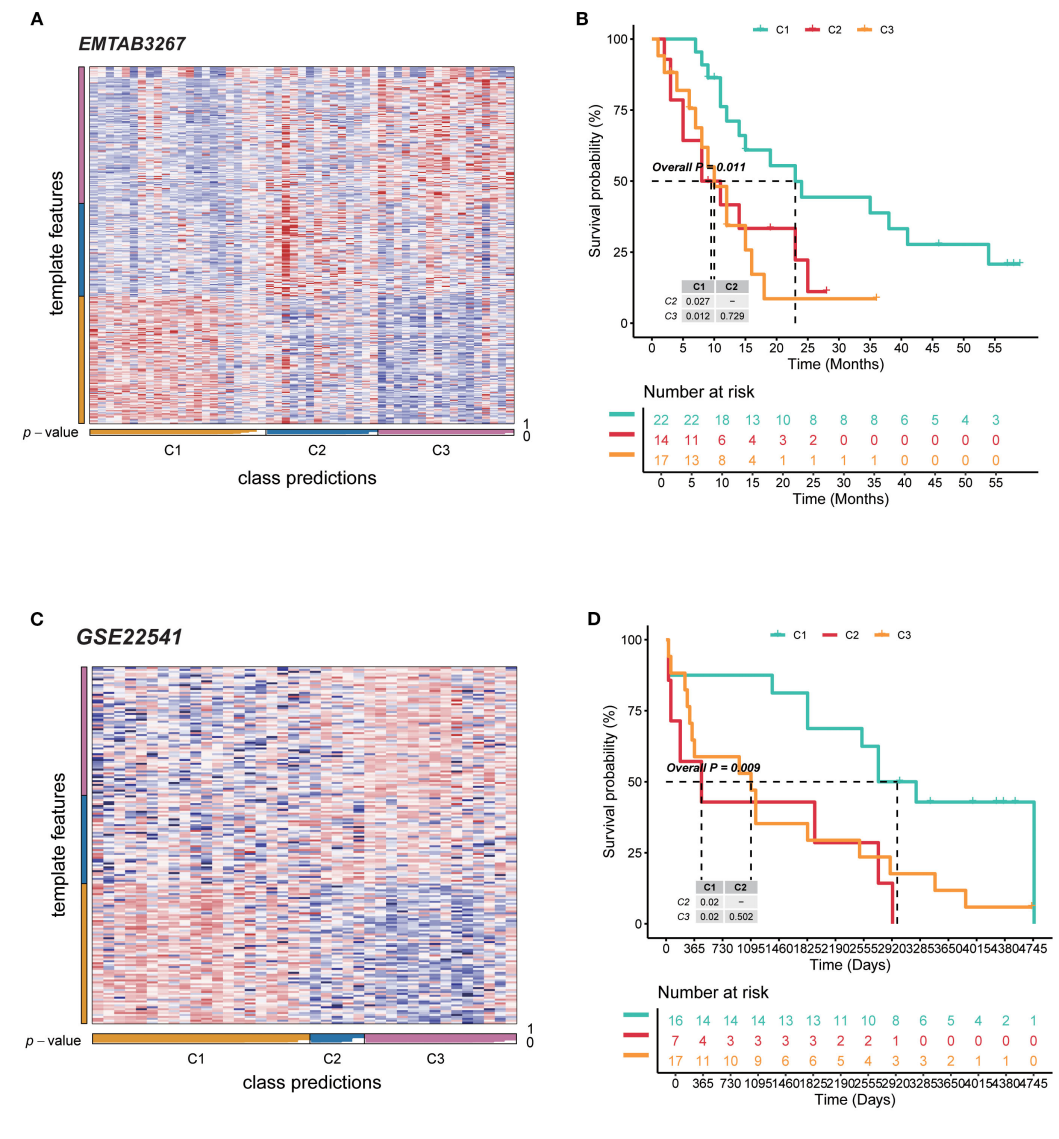
Validation of independent datasets revealed diverse outcomes of RCC patients. **(A)** NTP heatmap of the EMTAB3267 database for the reliability of three subtypes. **(B)** Kaplan–Meier curves for survival probability of three subtypes in EMTAB3267. **(C)** NTP heatmap of the database for the reliability of three subtypes. **(D)** Kaplan–Meier curves for survival probability of three subtypes in GSE22541. RCC, renal cell carcinoma; NTP, nearest template prediction.

For the external datasets, NTPs were carried out to validate the classification approach and the value of prognosis. Regarding EMTAB3267, NTP analysis confirmed the reliability of metabolism C1 to C3 clusters. Overall, subtypes had high prediction confidence (**Figure 2A**). Furthermore, cluster C2 contained the worst survival probability, cluster C1 had the best, and cluster C3 was in between (**Figure 2B**). For GSE22541, almost the same consequences were demonstrated as before (**Figures 2C, D**). To sum up, these three clusters possess precise prediction; even though there was no statistical significance between clusters C2 and C3, the entire tendency was consistent and accurate.

### GO and KEGG pathway enrichment analyses disclosed related functions and pathways

**Figure 3:**
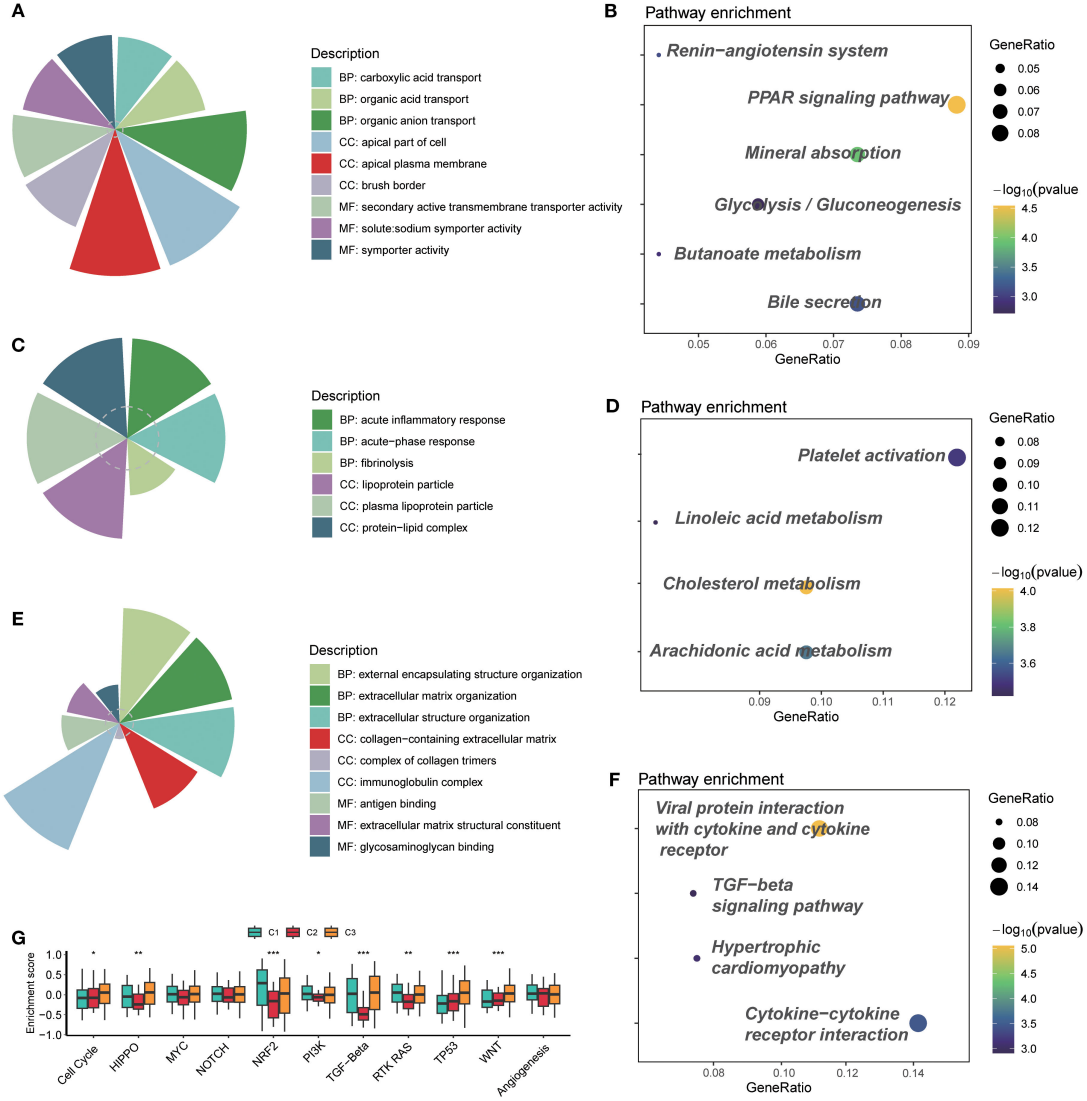
GO and KEGG pathway enrichment analyses disclosed related functions and pathways. **(A)** GO analysis of cluster C1. **(B)** KEGG pathway analysis of cluster C1. **(C)** GO analysis of cluster C2. **(D)** KEGG pathway analysis of cluster C2. **(E)** GO analysis of cluster C3. **(F)** KEGG pathway analysis of cluster C3. **(G)** Enrichment of typical signaling pathways across three subtypes. GO, Gene Ontology; KEGG, Kyoto Encyclopedia of Genes and Genomes. ns, not significant; *p < 0.05; **p < 0.01; ***p < 0.001.

For the C1 subtype, GO analysis revealed that BP included carboxylic acid transport, organic acid transport, and organic anion transport; CC included apical part of cell, apical plasma membrane, and brush border; MF included secondary active transmembrane transporter activity, solute:sodium symporter activity, and symporter activity (**Figure 3A**). KEGG pathway analysis found that the Peroxisome proliferators-activated receptor (PPAR) signaling pathway and mineral absorption were enriched significantly (**Figure 3B**). For the GO analysis of C2, acute inflammatory response, lipoprotein particle, and protein–lipid complex were observed (**Figure 3C**). KEGG revealed significantly enriched platelet activation, cholesterol metabolism, and arachidonic acid metabolism (**Figure 3D**). C3 mainly contained external encapsulating structure organization, immunoglobulin complex, antigen binding, etc., on GO analysis (**Figure 3E**); viral protein interaction with cytokine and cytokine receptor, and cytokine–cytokine receptor interaction were the most crucial pathways enriched by KEGG (**Figure 3F**).

Additionally, the evaluation of some classical signaling pathways suggested that cluster C2 represented a distinct difference compared to the other two subtypes. In particular, HIPPO, NRF2, PI3K, TGF-β, and RTK RAS were the typical pathways (**Figure 3G**). Among them, cluster C2 had low enrichment scores, which possibly means that genes in cluster C2 lacked response to pathway-related treatments, so the worst prognosis occurred.

### Clusters C2 and C3 involved an enabled immune microenvironment and implicated immunotherapy response

All three clusters indicated the outcomes of corresponding patients, and prostaglandin biosynthesis of cluster C2 and prostanoid biosynthesis of cluster C3 were of particular attention. The prostanoids are several lipid metabolites generated from 20-carbon fatty acids, and they play a key role in the inflammatory response *in vivo* (34). Recent studies have shown that prostaglandins and other members could regulate the activities of T cells, B cells, and cytokines in the tumor immune microenvironment, which causes immune exhaustion, and the tumor cell apoptosis was inhibited (35). RCC patients frequently encounter resistance to immunotherapy; therefore, we emphasized the relationship between clusters and the immune microenvironment of RCC.

**Figure 4:**
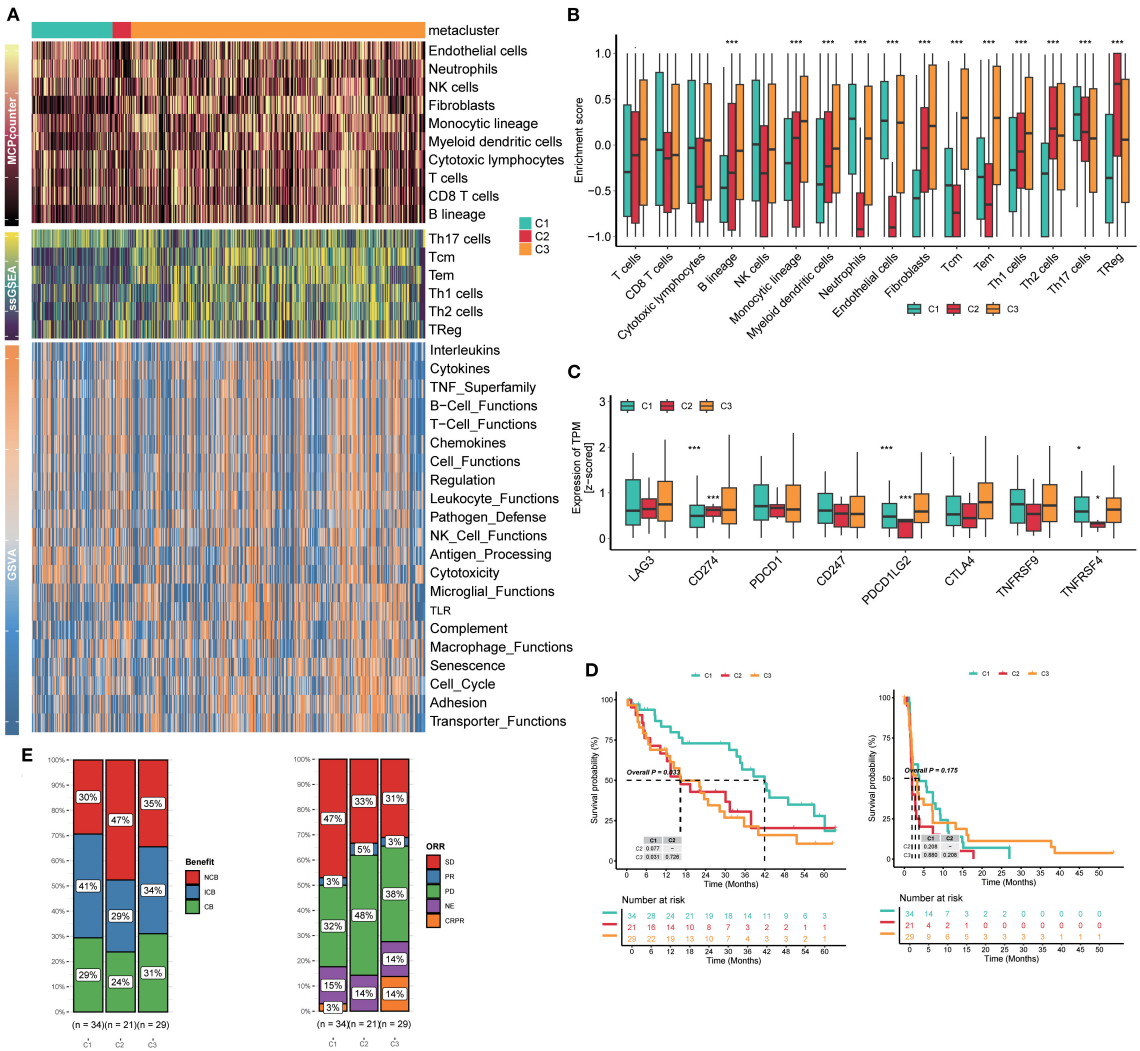
Clusters C2 and C3 involved an enabled immune microenvironment and implicated immunotherapy response. **(A)** Heatmaps of GSVA, ssGSEA, and MCP-counter analyses for three subtypes. **(B)** Enrichment of immune cells in three subtypes. **(C)** Enrichment of immune checkpoints in three subtypes. **(D)** Kaplan–Meier curves for survival probability of three subtypes. **(E)** Evaluation of the response to immunotherapy of three subtypes based on the CheckMate immunotherapy cohort. GSVA, gene set variation analysis; ssGSEA, single-sample gene set enrichment analysis; MCP-counter, Microenvironment Cell Populations-counter.

GSVA delineated clear immunophenotypic segregation among the three defined subtypes. Notably, C2 was characterized by a marked enrichment of immunosuppressive cellular populations, including interleukins, cytokines, B-cell functions, T-cell functions, NK cell functions, antigen processing, and macrophage functions; C3 displayed a relatively balanced TIME (**Figure 4A**). MCP-counter validated quantitative enrichment of suppressive cell populations in C2, which contained a higher portion of endothelial cells, neutrophils, NK cells, T cells, CD8 T cells, and so on (**Figure 4A**). In contrast, C1 was characterized by nearly absent immune infiltrates. Concerning samples in clusters, ssGSEA was conducted to explore the relative abundance of T cells. Consistent with GSVA findings, ssGSEA-derived enrichment scores reinforced the TIME dichotomy among the three subtypes: C2 and C3 involved more quantities in Th17, Tcm, Tem, Th1, Th2, and Treg cells (**Figure 4A**). A few immune-suppression cells, like Th1, Th2, and Treg cells, were highly enriched in clusters C2 and C3, while immune-activation cells, such as neutrophils and Th17 cells, were poorly enriched (**Figure 4B**), suggesting the poor outcomes of C2 and C3, which were consistent with the immune status.

Immune checkpoints are tightly linked to the clinical efficacy of RCC patients, and RCC is regarded as a kind of “hot” immunological tumor, so we explored the association between three subtypes and the expression of eight immune checkpoints. The results showed that cluster C2 exhibited a higher expression of CD274 than clusters C1 and C3; however, PDCD1LG2, TNFRSF9, and TNFRSF4 were at extremely low levels compared to the others (**Figure 4C**). We analyzed the downregulation of co-stimulatory molecules such as TNFRSF and TNFRSF4 in the C2 subtype, which may weaken T-cell activation and immune effects in the tumor microenvironment, thereby partially explaining why the immune efficacy remains poor despite high PD-L1 expression.

We also retrospectively reviewed all patients’ outcomes after accepting immunotherapy in the CheckMate immunotherapy cohort. RCC patients in cluster C1 achieved significantly prolonged survival compared to patients in C2 and C3 (*p*=0.033); the trend favored C1, suggesting durable immune engagement (**Figure 4D**). In particular, 47% patients in cluster C2 showed NCB, far more than those in C1 (30%) and C3 (35%). The frequency of CB in C2 was 24%, which was lower than that in C1 (29%) and C3 (31%). Meanwhile, the ORRs were similar to the previous benefits. Of patients in cluster C2, 48% were PD, but only 32% in C1 and 38% in C3 (**Figure 4E**). Hence, this classification is meaningful to patients’ effects of immunotherapy.

To strengthen the relation between subtypes and TLS, we calculated and explored TLS score, GC B, Tfh, FDC, Plasma, and B cell_score features among RCC patients, and the results showed that C3 presented higher expression of these TLS-related features, including CR2, FCER2, PAX5, CD19, MZB1, JCHAIN, DERL3, CCL19, CCL21, CXCL13, and BLC6. Furthermore, the C3 cluster also presented high enrichment scores of TLS score, GC B, Tfh, FDC, Plasma, and B cell_score features, indicating a high TLS formation tendency (**Supplementary Figure S2**).

### Drug sensitivity exhibited significant heterogeneity across three subtypes

Dozens of IC_50_ estimates were derived for each of the drugs from independent replicates. The distribution of IC_50_ values was visualized using violin–box hybrid plots to assess both the central tendency and variability across cell subtypes for each drug. Notable variability in drug sensitivity was observed across the three subtypes, with specific subtypes demonstrating distinct patterns of drug resistance or susceptibility.

**Figure 5:**
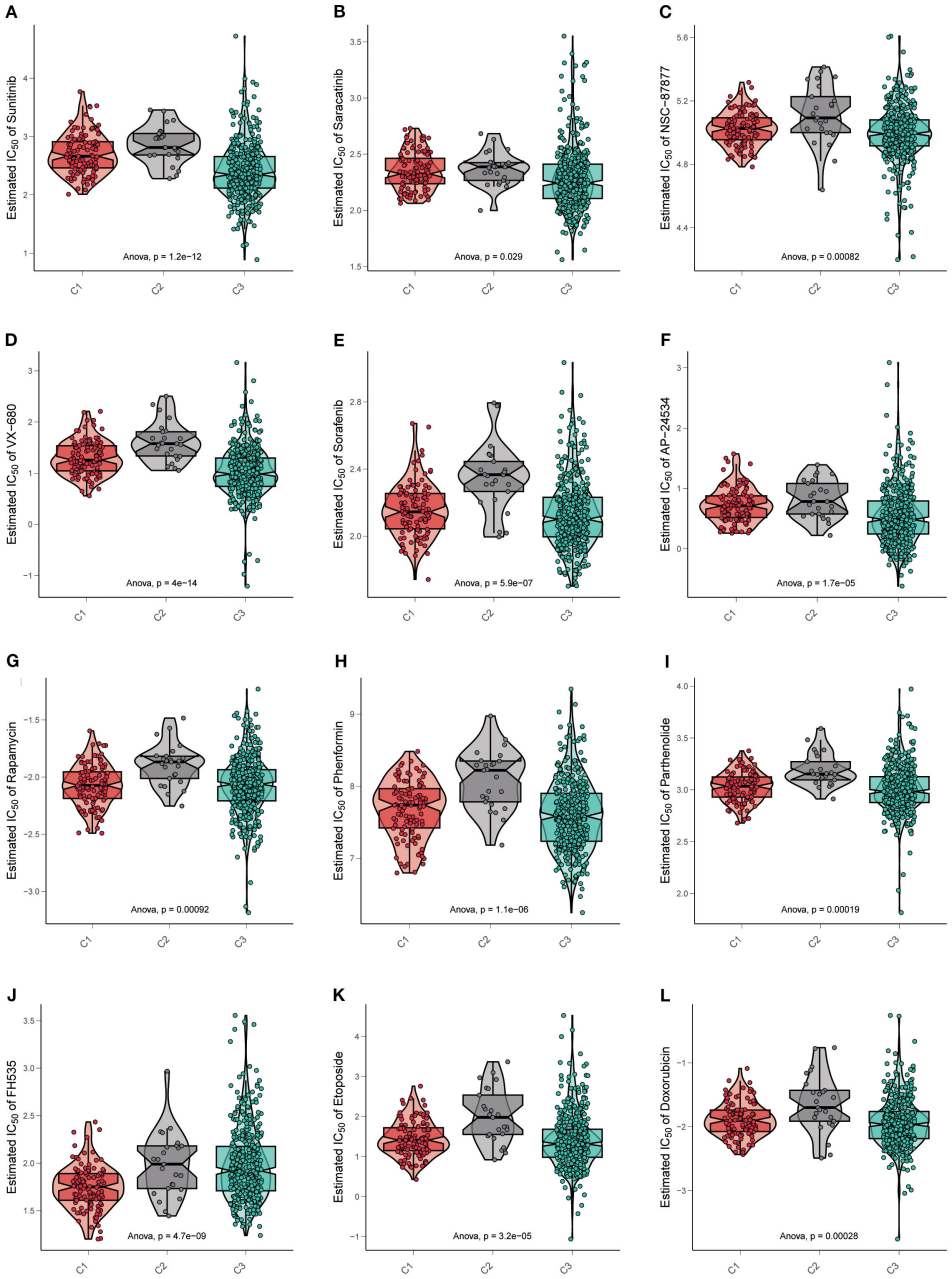
Drug sensitivity exhibited significant heterogeneity across three subtypes. **(A–L)** Violin plots display the estimated IC_50_ of multiple kinds of targeted drugs in three subtypes. Data are mean ± SD; statistical analysis was performed using one-way ANOVA.

Many kinds of drugs showed antitumor properties; herein, certain representative drugs were chosen. For the most frequently targeted medicine, TKI was observed to have a significant sensitivity gradient among the three subtypes. For the receptor TKI sunitinib, cluster C2 exhibited the highest estimated IC_50_ value, indicating resistance. One-way ANOVA confirmed that the observed differences in sunitinib sensitivity were statistically significant (*p* =1.2e−12) (**Figure 5A**). Saracatinib was an effective Src inhibitor, NSC-87877 was a Shp inhibitor, and both were non-receptor TKIs. Cluster C2 appeared to have the highest IC_50_ value as well (saracatinib *p*=0.029, NSC-87877 *p*=0.00082) (**Figures 5B, C**).

Other kinds of kinase inhibitors were committed to lethal effects on cancer cells. For the serine/threonine kinase inhibitors, such as VX-680, which were a part of evolutionary conserved families (Aurora-A, Aurora-B, and Aurora-C), controlling events of cell cycles, cluster C2 possessed a higher IC_50_ value than others (*p*=4e−14) (**Figure 5D**). Furthermore, multi-kinase inhibitors like sorafenib (*p*=5.9e−07) and AP24534 (*p*=1.7e−05) were also resistant to cluster C2 (**Figures 5E, F**).

Except for kinase targets, inhibitors specific to diverse pathways were considered beneficial in the extinction of cancer cells. Therefore, some drugs were estimated in three subtypes. Rapamycin and phenformin blocked the mTOR signaling pathway, inducing cancer cell autophagy and apoptosis, respectively. In cluster C2, both showed high IC_50_ values (rapamycin *p*=0.00092, phenformin *p* =1.1e−06) (**Figures 5G, H**). As a Nuclear Factor-κB (NF-κB) inhibitor, parthenolide was resistant in cluster C2 (*p*=0.00019) (**Figure 5I**), as well as the FH535, referred to as a Wnt/β-catenin pathway inhibitor (*p*=4.7e−09) (**Figure 5J**).

Otherwise, chemotherapeutics for cancers that target DNA replication are always available. Etoposide and doxorubicin were the common interventions inhibiting topoisomerase-II, thereby terminating DNA replication in cancer cells. As expected, drug resistance was the same in all the above drugs (etoposide *p* =3.2e−05, doxorubicin *p*=0.00028) (**Figures 5K, L**).

The overall drug response patterns corroborate our hypothesis that the molecular and phenotypic profiles of C1, C2, and C3 contribute to distinct therapeutic vulnerabilities. In particular, the cluster C2 phenotype appeared to be associated with enhanced drug resistance, which was in line with the poor prognosis of cluster C2 as mentioned before.

### Prostaglandin expression defines distinct prognostic and immunotherapy responses

**Figure 6:**
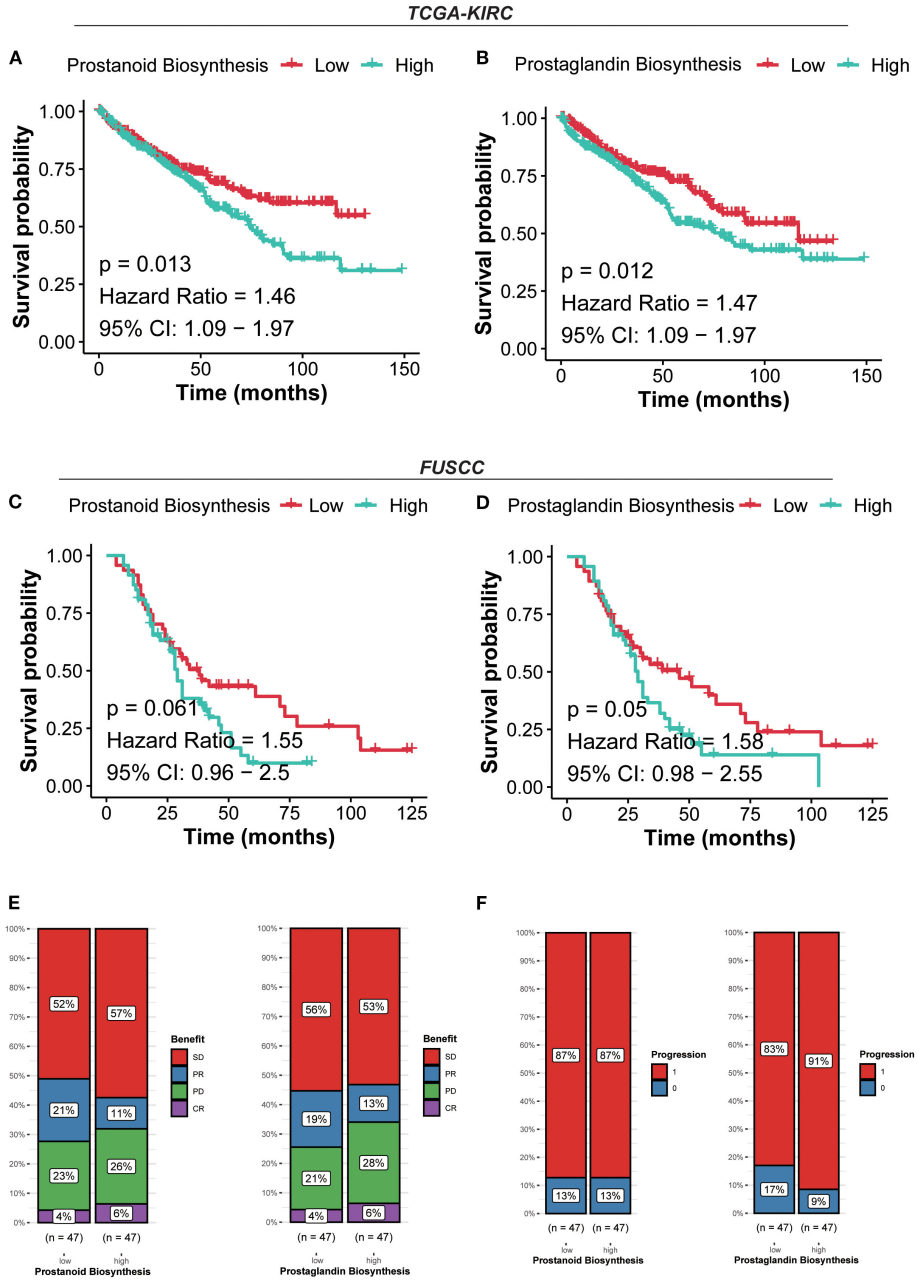
Prostaglandin expression defines distinct prognostic and immunotherapy responses. **(A)** Kaplan–Meier curves for survival probability of prostanoid biosynthesis in the TCGA-KIRC cohort. **(B)** Kaplan–Meier curves for survival probability of prostaglandin biosynthesis in the TCGA-KIRC cohort. **(C)** Kaplan–Meier curves for survival probability of prostanoid biosynthesis in the FUSCC cohort. **(D)** Kaplan–Meier curves for survival probability of prostaglandin biosynthesis in the FUSCC cohort. **(E)** Evaluation of the response to immunotherapy of prostanoid/prostaglandin biosynthesis based on the CheckMate immunotherapy cohort. **(F)** Evaluation of the progression of prostanoid/prostaglandin biosynthesis based on the CheckMate immunotherapy cohort.

For further validation of cohorts, patients were stratified into high and low prostanoid/prostaglandin expression groups based on the median expression of the metabolism gene signature (PGE2). In the TCGA-KIRC cohort, patients with high prostanoid/prostaglandin expression exhibited significantly worse survival (prostanoid *p*=0.013, HR=1.46, 95% CI: 1.09–1.97; prostaglandin *p*=0.012, HR=1.47, 95% CI: 1.09–1.97). Similarly, in the FUSCC cohort, patients with high prostanoid/prostaglandin expression had worse survival (prostanoid *p*=0.061, HR=1.55, 95% CI: 0.96–2.5; prostaglandin *p*=0.05, HR=1.58, 95% CI: 0.98–2.55) (**Figures 6A–D**).

Patients in the high prostanoid group with PD were more than those in the low prostanoid group (26% vs. 23%), the same as in the high/low prostaglandin group (28% vs. 21%). In terms of progression, the high prostanoid and low prostanoid groups showed no difference (87% vs. 87%), but patients in the high prostaglandin group were more likely to progress than others (91% vs. 83%) (**Figures 6E, F**). These results suggested that high prostanoid/prostaglandin RCC harbors a more suppressive and therapy-resistant microenvironment, with lower immunogenicity and metabolic reprogramming.

### Prostaglandin biosynthesis pathway regulates RCC progression and prognosis

To evaluate the clinical relevance of prostaglandin biosynthesis in the context of therapeutic response, immunohistochemistry (IHC) analysis was performed on RCC tissue microarrays (TMAs) obtained from patients with differential responses to targeted therapy. A total of 12 tumor specimens were analyzed and stratified into four groups according to clinical response criteria: CR (n = 3), PR (n = 3), SD (n = 3), and PD (n = 3). For analytical purposes, CR and PR were classified as the non-progressive disease (non-PD) group, while SD and PD were considered PD based on radiological and clinical progression metrics.

**Figure 7:**
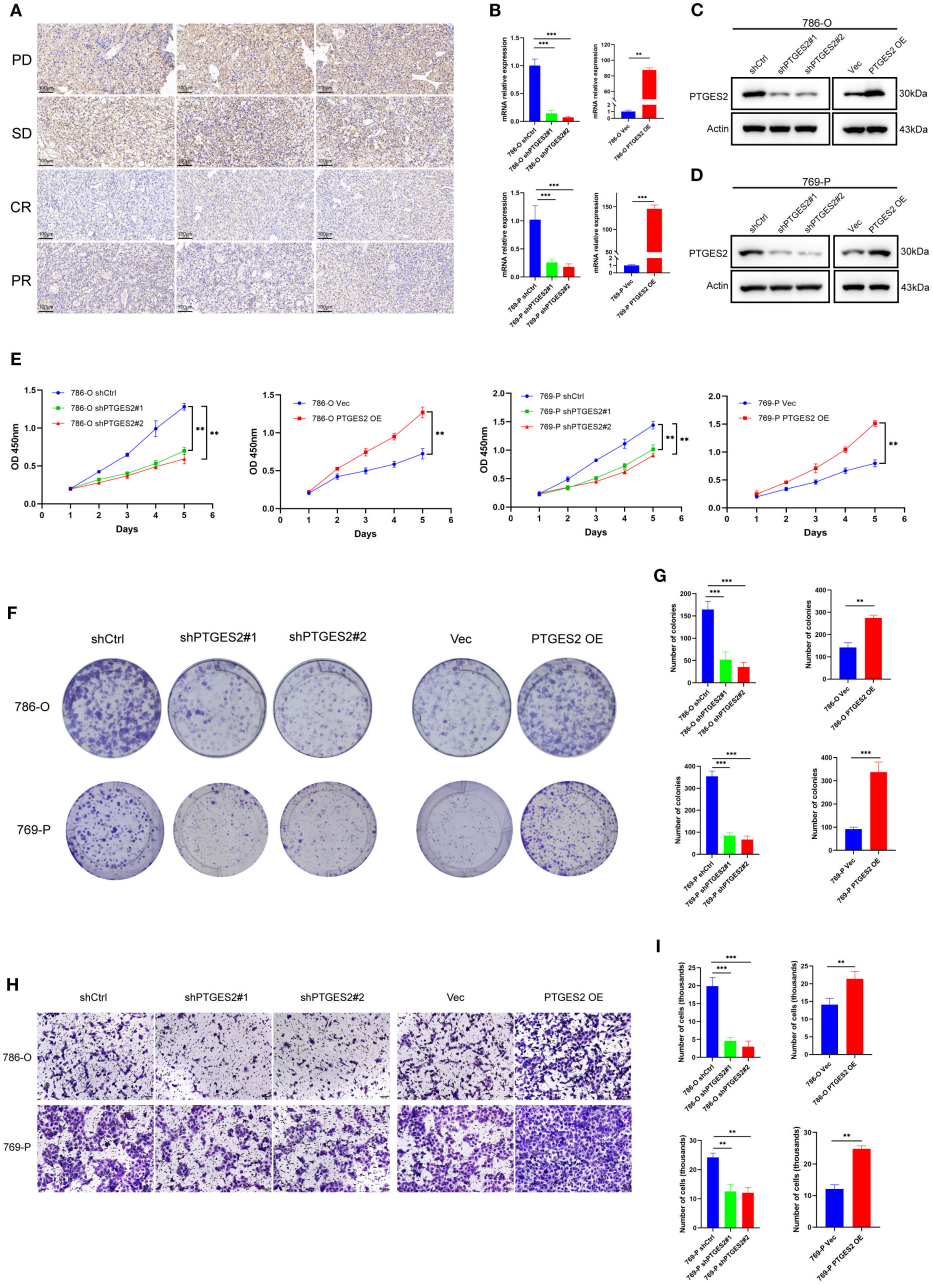
Prostaglandin biosynthesis pathway regulates RCC progression and prognosis. **(A)** PTGES2 expression was analyzed by immunohistochemistry in tumor tissues of patients with different outcomes. Scale bars, 100 μm. **(B)** Validation of knockdown and overexpression of PTGES2 in RCC cell lines by qPCR. **(C)** Validation of knockdown and overexpression of PTGES2 in 786-O cell line by WB. **(D)** Validation of knockdown and overexpression of PTGES2 in 769-P cell line by WB. **(E)** CCK-8 assays showed the influence of PTGES2 on cell proliferation. **(F)** Colony formation assays showed the influence of PTGES2 on cell proliferation. **(G)** Statistics of colonies showed the difference after PTGES2 was abnormally expressed. **(H)** Transwell assays showed the influence of PTGES2 on cell migration. Scale bars, 100 μm. **(I)** Statistics of transmembrane cells showed the difference after PTGES2 was abnormally expressed. Data are mean ± SD; statistical analysis was performed using one-way ANOVA. ns, not significant; *p < 0.05; **p < 0.01; ***p < 0.001. RCC, renal cell carcinoma; WB, Western blotting.

Here, we found prostaglandin E synthase 2 (PTGES2), one key enzyme catalyzing the synthesis of prostaglandin E2, which was associated with the progression of some tumors, such as colorectal cancer (CRC), hepatocellular carcinoma (HCC), and RCC (36–38). IHC staining revealed a distinct expression pattern of PTGES2 across the four groups. In the PD subgroup (PD and SD), PTGES2 exhibited markedly higher expression, characterized by strong staining intensity in RCC cells (**Figure 7A**). In contrast, the non-PD subgroup (CR and PR) demonstrated weak-to-moderate PTGES2 staining, with a substantial reduction in both staining intensity and percentage of positive cells. These findings suggested that PTGES2 expression correlated positively with therapy resistance and tumor progression status in RCC, which was in accordance with cluster C2. In addition, PTGES2 was inversely associated with TLS programs: TLS (*r* = −0.17, *p* =6e−5), GC B (*r* = −0.31, *p* = 3.6e−13), and Plasma (*r* = −0.26, *p* = 3.4e−9); FDC showed no association (*r* = −0.054, *p*=0.22); Tfh showed a weak positive correlation (*r*=0.15, *p* =6e−4) (**Supplementary Figure S3A**). Consistently, high PTGES2 tumors had lower TLS features (all *p* ≤ 0.05), with no change in FDC (*p*=0.59) and only a marginal increase in Tfh (*p*=0.075) (**Supplementary Figure S3B**), indicating that high PTGES2 is linked to reduced TLS enrichment. Across six deconvolution methods, high PTGES2 tumors consistently exhibited increased Tregs and M2-like macrophages with relative depletion of CD8+ T cells and NK cells (**Supplementary Figure S4A**). Interferon-α/γ responses, cytokine–cytokine receptor interaction, and immune system signaling were enriched in high PTGES2 (**Supplementary Figure S4B**), consistent with an inflamed yet functionally suppressive microenvironment. We observed modest negative associations with PD-L1 and CTLA-4, a weak positive association with TIGIT, and no material association with PD-1 or LAG-3 (**Supplementary Figures S4C, D**).

To further investigate the role of PTGES2 in RCC cell proliferation and metastasis, we performed a series of *in vitro* assays following the stable knockdown and overexpression of PTGES2 in the 786-O and 769-P cell lines. The efficiencies of the knockdown and overexpression of PTGES2 were verified at both the mRNA and protein levels by quantitative polymerase chain reaction (qPCR) and Western blotting (WB) analysis, respectively. The results showed that PTGES2 was successfully knocked down and overexpressed in both 786-O and 769-P cells (**Figures 7B–D**).

The CCK-8 assay was used to explore cell proliferation. Knockdown of PTGES2 resulted in a significant reduction in cell reproductive capacity in both 786-O and 769-P cell lines; in contrast, PTGES2 overexpression contributed to the obvious proliferation of RCC cells, and all differences were statistically significant (*p* < 0.05) (**Figure 7E**). The colony formation capability of PTGES2 knockdown cells was significantly decreased compared to that of shCtrl cells (**Figure 7F**), the colony counts in the shPTGES2 groups were decreased under the microscope, and the difference was statistically significant (*p* < 0.05). The results were reversed while PTGES2 was overexpressed in RCC cell lines (**Figures 7F, G**). These data indicate that PTGES2 can promote the proliferation of RCC cells.

To evaluate the metastasis of PTGES2 in RCC cells, a Transwell assay was conducted to determine the cell migration ability. The results suggested that shPTGES2 cells exhibited significantly reduced migration compared to the control cells (**Figure 7H**). The number of migrated cells was decreased visibly in shPTGES2-treated cells after cell counting, and the difference was statistically significant (*p* < 0.05) (**Figure 7I**). Certainly, PTGES2 overexpression was observed to motivate the migration of RCC cells, and the difference between the vector and overexpression groups was statistically significant as well (*p* < 0.05) (**Figures 7H, I**). These results revealed that PTGES2 drove RCC cell migration and may be positive for the metastasis of RCC.

In general, these *in vitro* experiments strengthened the association between PTGES2 expression and clinical response. Our observations implicated the translational value of PTGES2, which could serve as a potential biomarker and a therapeutic target for RCC.

## Discussion

There has been sufficient evidence to prove that RCC is a highly heterogeneous and metabolic disease, and the Warburg effect produces abundant energy to support the metabolism and unlimited growth of cancer cells (39). Next-generation sequencing indicates that the VHL mutation exists in almost 90% of RCC cases, following the accumulation of HIF, which enables cells to adapt the glycolysis in the microenvironment. As the tumor becomes more advanced, this phenomenon appears more obviously (40). In the past few decades, many key oncogenes have been discovered to change metabolic homeostasis to facilitate cancer cell growth (41, 42). Multi-omics technology has ushered in a new era, precise treatment comes to a great innovation, and reliable subtype identification seems crucial. Currently, research on analyzing the relationship between tumor molecular subtypes, immune microenvironment, and treatment sensitivity by integrating multi-omics data is highly topical (43, 44). At present, proteomics based on epigenetic regulation seems to elucidate some basic principles of RCC, DNA, RNA, or histone modifications involved in the altered metabolism in RCC to some degree (45). Genomics and transcriptomics can also recognize some molecular biomarkers and relative subtypes (46). Although these classifications can be meaningful, a specialized system directly aiming at RCC metabolism is deficient. Herein, by integrating and analyzing metabolic genes in three datasets—TCGA, EMTAB3267, and GSE22541—we divided RCC into clusters C1, C2, and C3. We conducted a cross-comparison between the metabolic subtypes and the RCC molecular subtypes proposed by TCGA (including ClearCell A/B), as well as other transcriptomic features. The analysis results indicated that there was only partial overlap between the proposed metabolic subtypes and the TCGA classification. Additionally, the metabolic subtypes of some patients were completely inconsistent with the known molecular subtypes, suggesting that our metabolic classification provides complementary information to the traditional transcriptome-driven classifications (47).

Cluster C1 was primarily involved in fatty acid degradation and various amino acid metabolism, and patients had a better prognosis than those in C2 and C3. Actually, the metabolic abnormal pattern exhibited by cluster C1 has been observed in the analysis of various cancer types, suggesting that it may be a relatively common phenomenon in the process of tumor metabolic reprogramming (48). FAs are the necessary substrates for tumor survival; they can maintain the cell membrane’s stability and motivate signal transduction; in particular, RCC is abundant in lipid profile, and internal fatty acid metabolism appears to be highly active (49). RCC cells drive *de novo* FA synthesis to ensure continuous supply (50). During the entire biochemistry, various kinds of molecules, including acetyl-CoA, palmitate, stearoyl-CoA desaturase (SCD), and some other unsaturated fatty acids, have been proven to be the underlying targets of RCC (50–53). For instance, ATP citrate lyase (ACLY), one of the key enzymes in fatty acid metabolism, was considered to promote the proliferation and migration of RCC cells (54). Another enzyme, FASN, was found to be upregulated in RCC and stimulated tumor progression (55). Furthermore, HIFs control the RCC tumorigenesis in the same way (56). For the amino acid metabolism in RCC, the serine/glycine biosynthesis was one of the essential metabolic pathways, and serine hydroxymethyltransferase (SHMT) represented the central enzyme, associated with pathological grading and poor prognosis of RCC patients (57). In addition, as a non-essential amino acid, glutamine supplies materials for the TCA cycle and amidotransferase pathways in RCC, and some inhibitors that are specific to glutamine metabolism were put into effect, which led to modest growth suppression in RCC cells (58–60). Overall, cluster C1 displayed more about the general characteristics of metabolism-associated tumors, and FA metabolism and amino acid metabolism were complicated, but corresponding mechanisms were comparatively intensive; even a few therapeutic targets had been applied in clinical (61) studies, which may be a reason for the better prognosis.

For clusters C2 and C3, prostaglandin (PG) biosynthesis and prostanoid biosynthesis were enriched, respectively, and a worse prognosis than that in C1 occurred. PGs are nearly the same as prostanoids in nature. PGs have emerged as critical regulators in inflammation, cardiovascular disease, and tumors (62–64). Bioactive PGs contain PGI2, PGE2, PGF2α, PGD2, and thromboxane A2 (TxA2), which are synthesized from arachidonic acid by cyclooxygenase (COX) (65). We systematically compared the expression profiles of prostaglandin metabolism-related genes in the C2 and C3 clusters. The “prostaglandin biosynthesis” in C2 is mainly characterized by the activation of the PGE2 branch, reflecting that this subtype may primarily mediate immune suppression and tumor microenvironment remodeling through PGE2. In contrast, the “widespread” biosynthesis in C3 refers to the global activation of multiple prostaglandin branches, including but not limited to PGE, PGD, and PGF. Therefore, although the overall prostaglandin metabolism level in C3 is elevated, the coexistence of multiple branches weakens the adverse effects driven by a single branch (such as PGE2), thus resulting in an intermediate prognosis between C2 and other subtypes.

Previous studies have shown that prostanoid biosynthesis emerges as a cascade, massive proinflammatory cytokines were exploited, and they initiated downstream interferon, interleukin, lymphokine, chemokine, and tumor necrosis factor; sometimes, inflammation and immune response were overactivated, producing prostanoid-angiogenic response (66–68). In RCC, products of prostanoid biosynthesis are widely infiltrated into the tumor microenvironment, leading to tumor-associated angiogenesis, cell growth, and metastasis (69, 70). The overexpression of vascular endothelial growth factor (VEGF) and relevant VEGF receptor (VEGFR) has been proven to be pivotal in RCC angiogenesis (71), and antiangiogenic therapies have been employed. Sunitinib, one of the TKIs, is the first-line targeted medicine for advanced RCC, which inhibits the targets of VEGFR and angiogenesis, but sunitinib resistance is seriously increasing (72, 73). Multiple studies have confirmed that the abnormal activation of components in the VEGF pathway generates a compensatory mechanism, consequently contributing to the resistance (74–76). Luo et al. found that the obvious activation of the COX-2-PGE2 pathway in RCC cells promoted sunitinib resistance (77). As a result, extremely sophisticated factors of prostanoid biosynthesis act as a double-edged sword in RCC angiogenesis, suggesting a favorable but inscrutable target for treatments.

Moreover, prostanoid biosynthesis is tightly related to the immune microenvironment. Ahmadi et al. came up with a novel perspective on tumor immunology. They found that PGE2 prevented the maturation of dendritic cells (DCs), and the activation of naive CD8+ T cells was terminated; meanwhile, the CD8+ CD28− T cells were induced, and tumor cells could not be killed. The overexpression of COX-2 had a similar effect on DCs and prompted tumor cells to generate lymphatic metastasis (78). Many researchers have suggested that prostanoids exert profound functions of immune regulation. A noted experiment established melanoma models that are sensitive and resistant to immunotherapies. RNA-seq and metabolomics analysis revealed that the PGE2 involved in the prostaglandin synthesis pathway contributed to T-cell responses in the tumor microenvironment. *In vivo* immunologic research highlighted that the overexpression of PGE2 and reduced IFN-γ simultaneously led to immune escape in RCC (79). Except for the CD8+ T cells, PGE2 and its receptor EP2/EP4 additionally impaired the oxidative phosphorylation (OXPHOS) and c-MYC targets of M1-like macrophages, as well as ribosome biogenesis. Ultimately, the effect of immune exhaustion was noted (80). NK cells were considered the first line of defense against cancer and viral infections. They may be more critical than T cells in anti-metastatic immunity in cancers (81). PGE2 was secreted increasingly in disseminated tumor cells while metastasis happened; PGE2-EP2/EP4 modified the gene expression and caused dysfunction in NK cells, suppressing the key anti-metastatic cytokines (82). Inhibitors targeting PGE2 rescued NK cell function, the immune escape was overcome, and the NK cell-mediated killing of cancer cells was enhanced (83). Collectively, attributed to the mysteriously immune microenvironment, although the immunotherapies were implemented in advanced RCC patients, diversified prostanoids made unpredictable differences to the immunologic targets. Therefore, the overall prognosis was still not promising in cluster C2. PTGES2-driven PGE2 suppressed dendritic cell maturation and limited B/Tfh recruitment and TLS formation (84). Consistently, high PTGES2 tumors showed the lowest TLS enrichment. This PGE2-driven, TLS-deficient state may blunt antitumor immunity and Immune checkpoint blockade (ICB) benefit; targeting the COX-2/PTGES2–EP4 axis could help restore TLS programs.

To validate the precision of our model, a few RCC tissues were screened, which were from RCC patients treated with immunotherapies in the FUSCC cohort, and follow-up datasets were complete. Tumors in the “PD” group showed higher expression of PTGES2 than in the “non-PD” group. The synthesis of PGE2 was an extremely complicated process; one targeted metabolite profiling uncovered that PTGES2 was the key enzyme (85), and PTGES2 was confirmed to influence the survival of cancer cells (36, 37). For the CRC cells, while elevated PTGES2 led to PGE2 boosting, reactive oxygen species (ROS) were largely produced, and genomic instability was triggered, ultimately driving cancer progression (86). On this basis, we conducted *in vitro* assays to explore the biological function of RCC cells. The results highlighted the significance of PTGES2 in RCC; meanwhile, the mechanisms of prostanoid biosynthesis in RCC deserve intensive studies.

In summary, this original classification based on tumor metabolism provided new insights into RCC; all three clusters covered the majority of metabolic features of the tumor microenvironment. It was worth noting that cluster C1 represented more about the early stage of RCC, in which FA metabolism and amino acid metabolism were active, and the prognosis was better. Clusters C2 and C3 pointed to prostanoid biosynthesis, which was associated with immune exhaustion and indicated a poor prognosis. We integrated the public databases and our cohort, not only elucidating the clinical significance of three clusters but also emphasizing the importance of metabolism-related therapies in advanced RCC patients. However, we also recognize that there are still some potential or unknown confounding factors that have not been adequately controlled in this study, which may affect the interpretation of some of the research results. In particular, variables such as patient age, gender, underlying disease status, lifestyle, and treatments could all potentially act as confounding factors in the relationship between the exposure factors and outcomes in this study. Furthermore, there may be selection bias in the sample selection process of this study. For example, the included cases were mainly from public databases or specific centers. For the analysis of certain specific subgroups, the sample size was further reduced, which may have exacerbated the impact of this bias. At the same time, the total sample size of this study was relatively limited, resulting in insufficient overall statistical power, especially in the subgroup analysis or multivariate analysis. Therefore, we suggest that future studies with a larger sample size should be conducted in multiple centers and with higher-quality data to further confirm the findings of this study.

## Conclusion

In conclusion, this study classified RCC from the metabolic perspective and proposed three subtypes with different kinds of metabolism. Every cluster exhibited a specific metabolic status and appeared to have highly predictive value. Meanwhile, our classification may offer insights for improving the effects of immunotherapies in advanced RCC patients.

## Data Availability

The datasets presented in this study can be found in online repositories. The names of the repository/repositories and accession number(s) can be found in the article/**Supplementary Material**.
