# Supplementary material for: Comprehensive analysis of metabolic patterns in renal cell carcinoma: implications for prognosis and treatment
Source: Front Immunol. 2025 Sep 17;16:1630053. doi: 10.3389/fimmu.2025.1630053 (PMC12484182; doi:10.3389/fimmu.2025.1630053)

Supplementary Material
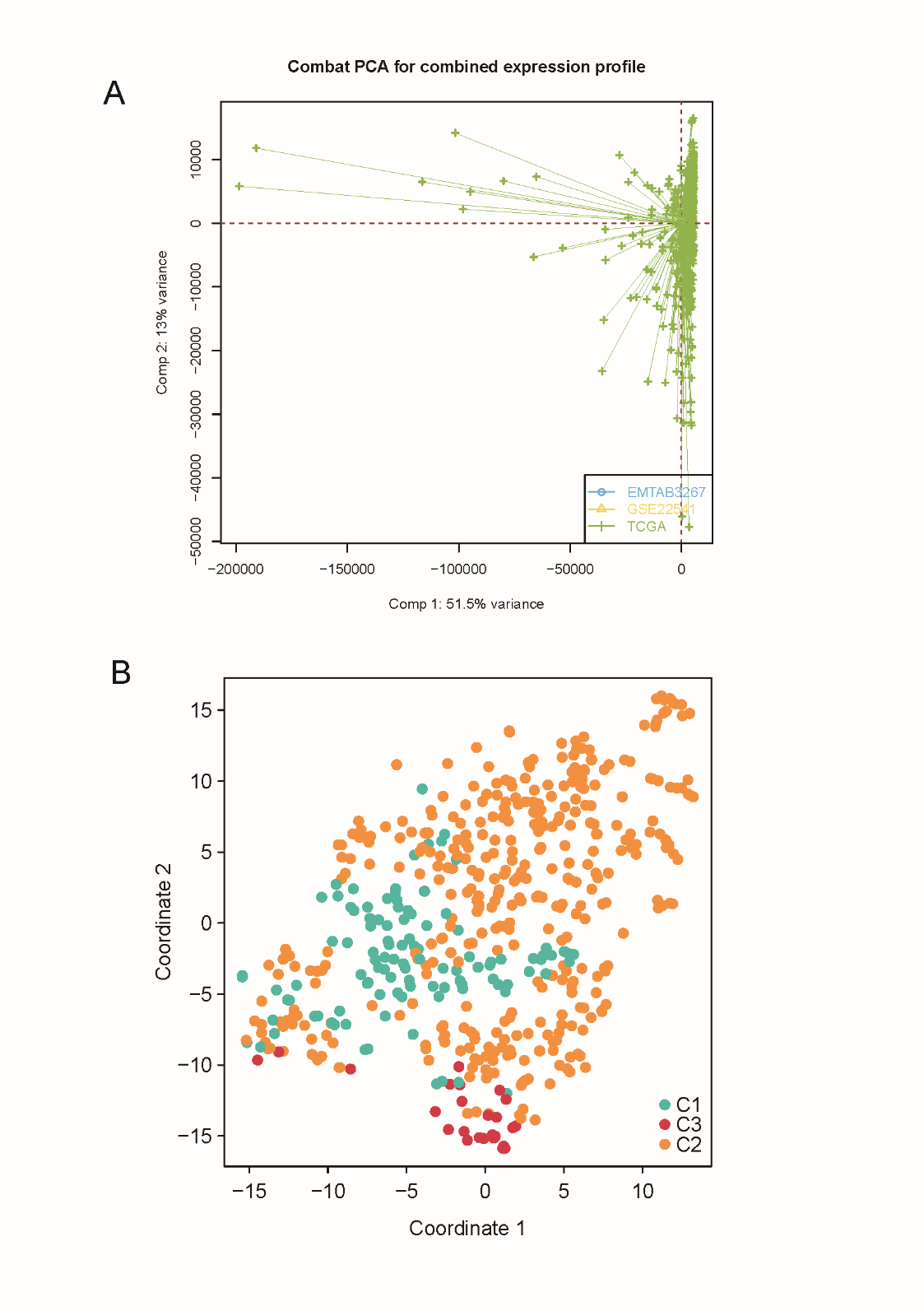


**Figure S1:** **Batch correction and subtype separation across combined cohorts. (A)** Principal component analysis (PCA) of the merged expression matrix after ComBat batch-effect adjustment. (**B**) Two-dimensional ordination of the same samples showing separation of the three metabolic subtypes (C1–C3) in feature space (coordinates 1 and 2).


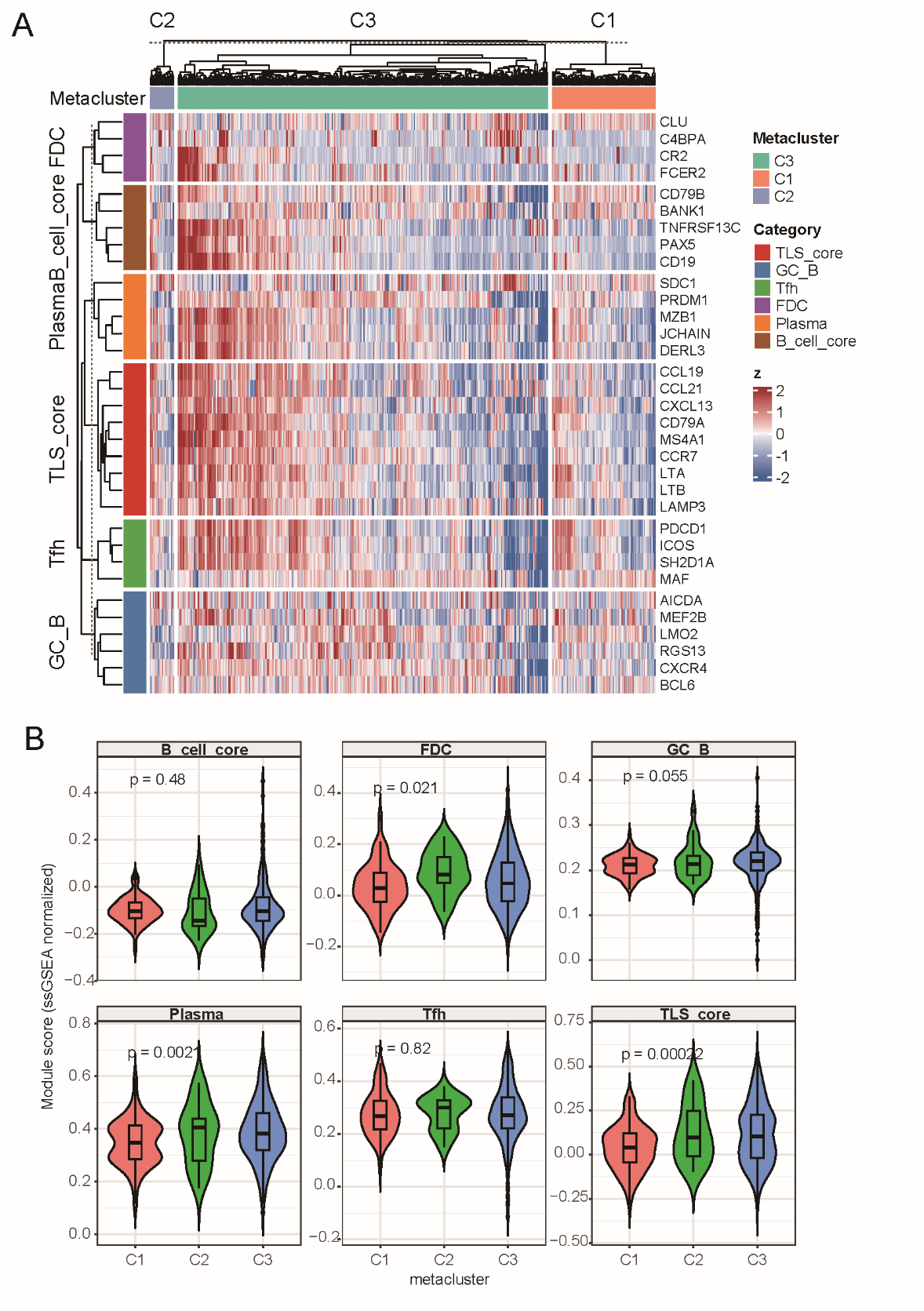


**Figure S2. TLS-related programs across metabolic subtypes. (A)** Heatmap of genes belonging to six TLS-related modules, including TLS score, GC B, Tfh, FDC, Plasma, and B cell in the TCGA-KIRC cohort. (**B**) Violin plots with overlaid boxplots showing ssGSEA-normalized module scores across C1/C2/C3.


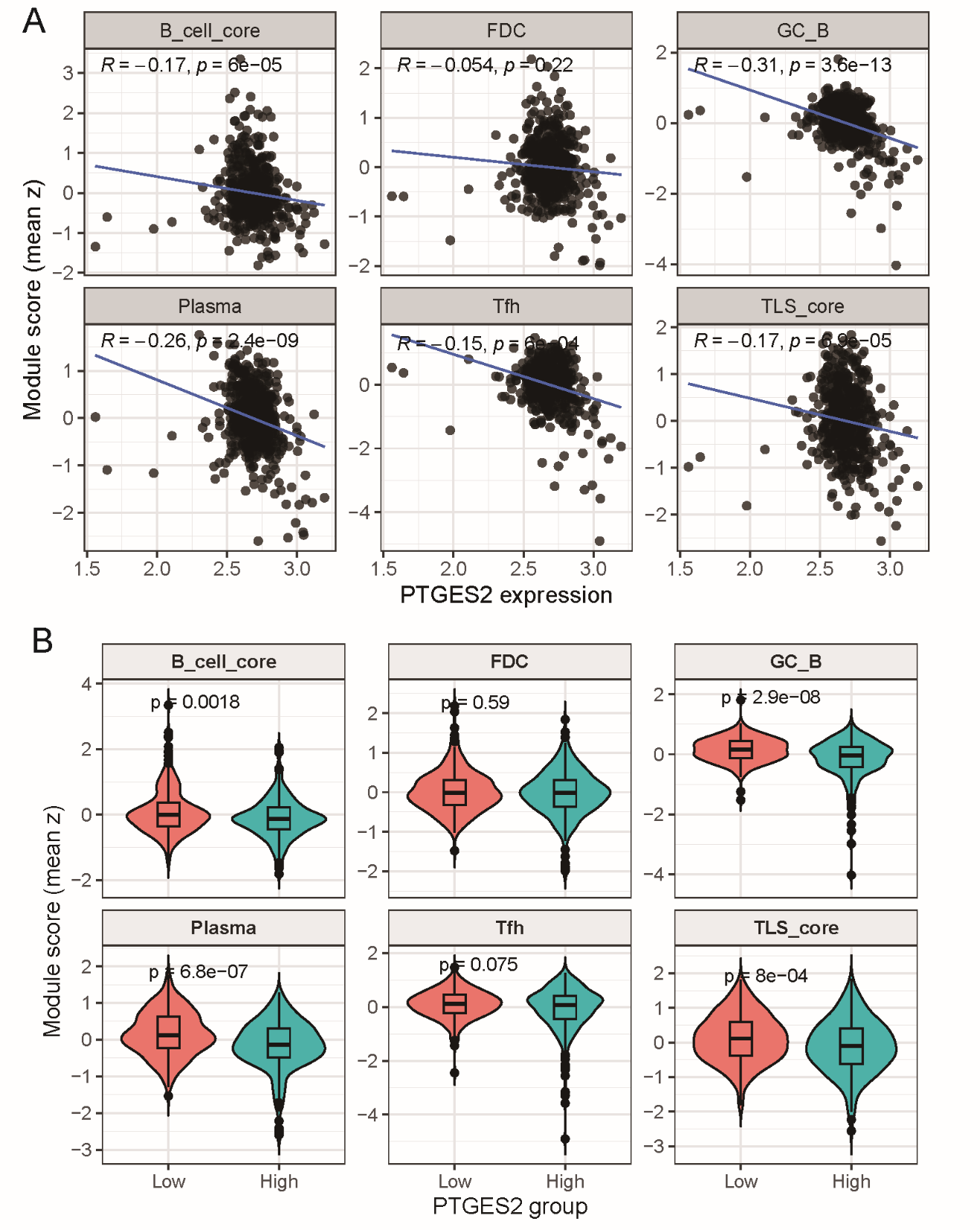


**Figure S3. Association between PTGES2 expression and TLS-related programs. (A)** The relationship between PTGES2 expression and module scores for six TLS-related programs (TLS score, GC B, Tfh, FDC, Plasma, and B cell). **(B)** Violin plots comparing module scores between PTGES2-low and PTGES2-high tumors (median split). *p* values are from two-sided Wilcoxon rank-sum test. TLS, tertiary lymphoid structure; GC_B, germinal-center B-cell module; Tfh, T follicular helper-cell module; FDC, follicular dendritic-cell.


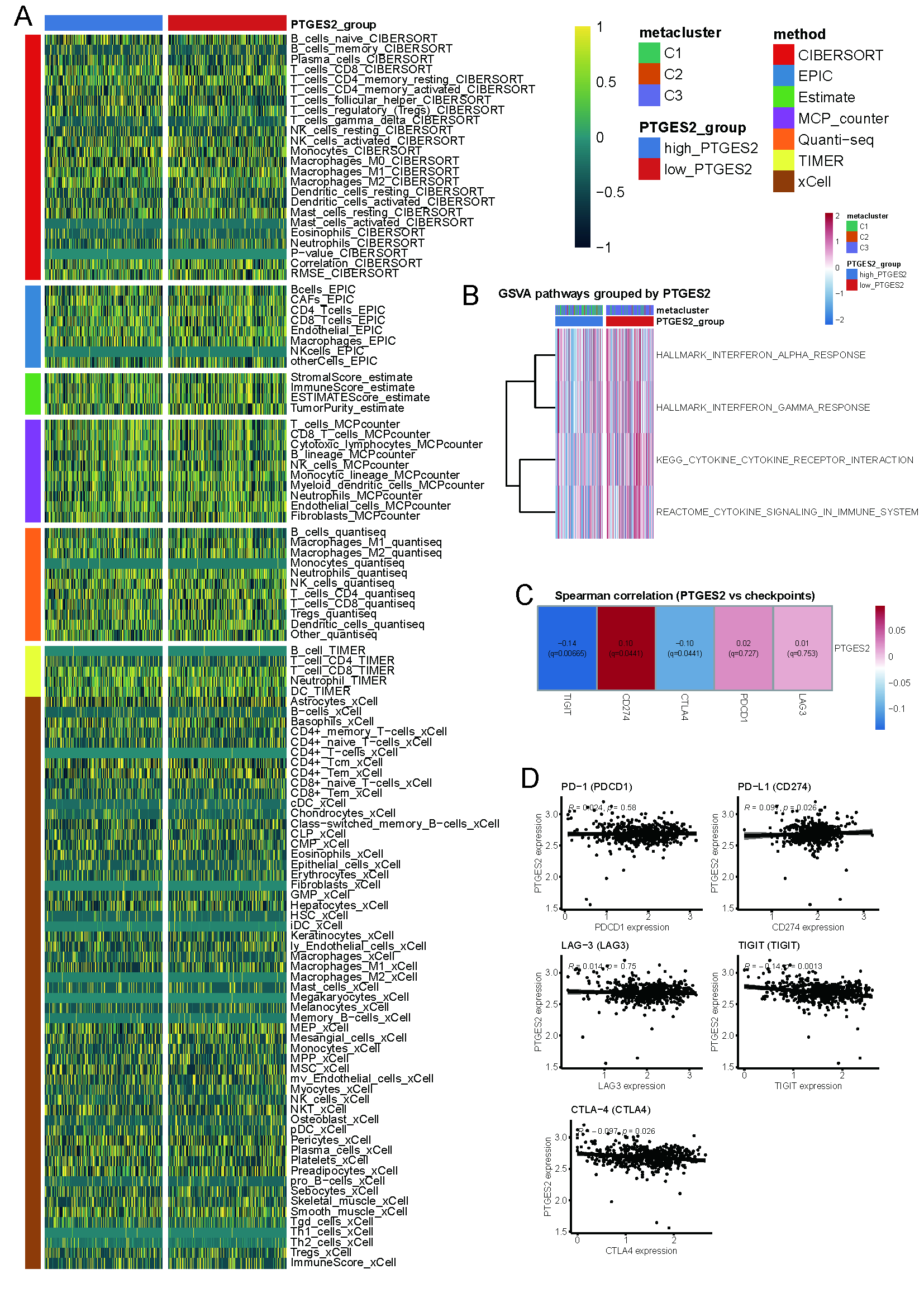


**Figure S4. Impact of PTGES2 and immunocytes and tumor immune contexture. (A)** Multi-algorithm immune-cell deconvolution across samples stratified by PTGES2 group, estimated by CIBERSORTx, EPIC, ESTIMATE, MCP-counter, quanTIseq, TIMER, and xCell. **(B)** GSVA comparing PTGES2-high vs. PTGES2-low tumors highlights enrichment. **(C)** Spearman correlations between PTGES2 and immune-checkpoint transcripts. **(D)** Spearman correlations between PTGES2 and immune-checkpoint expression.

**Supplementary materials 2**
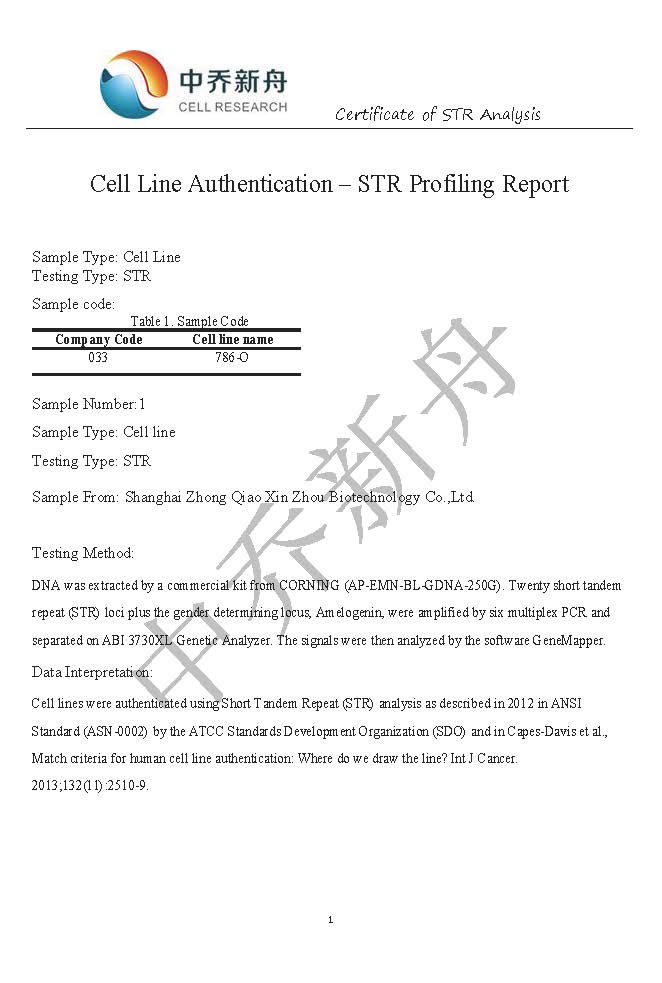


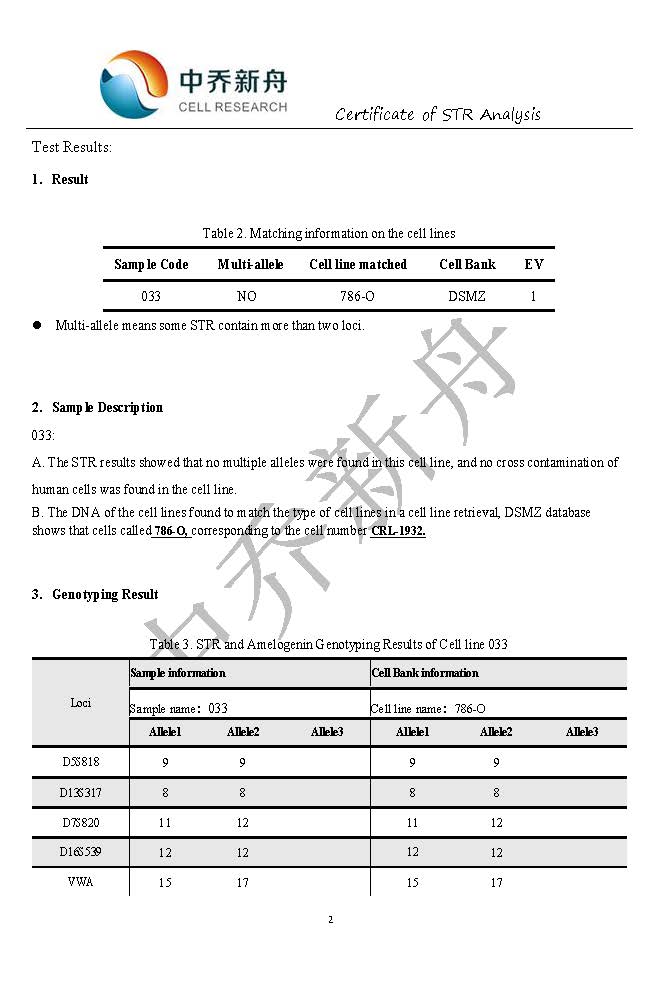

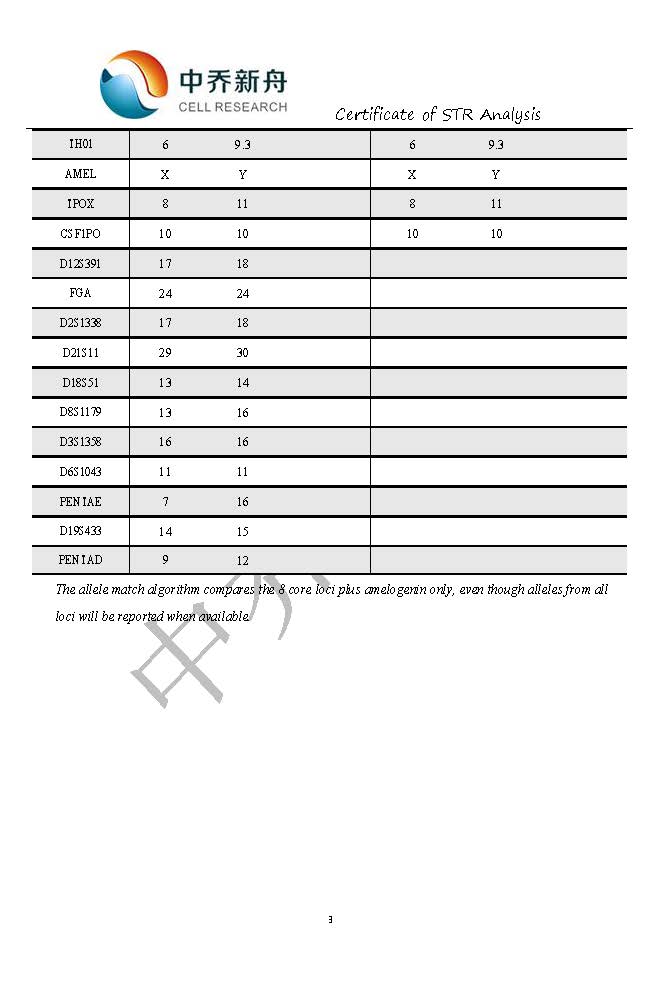

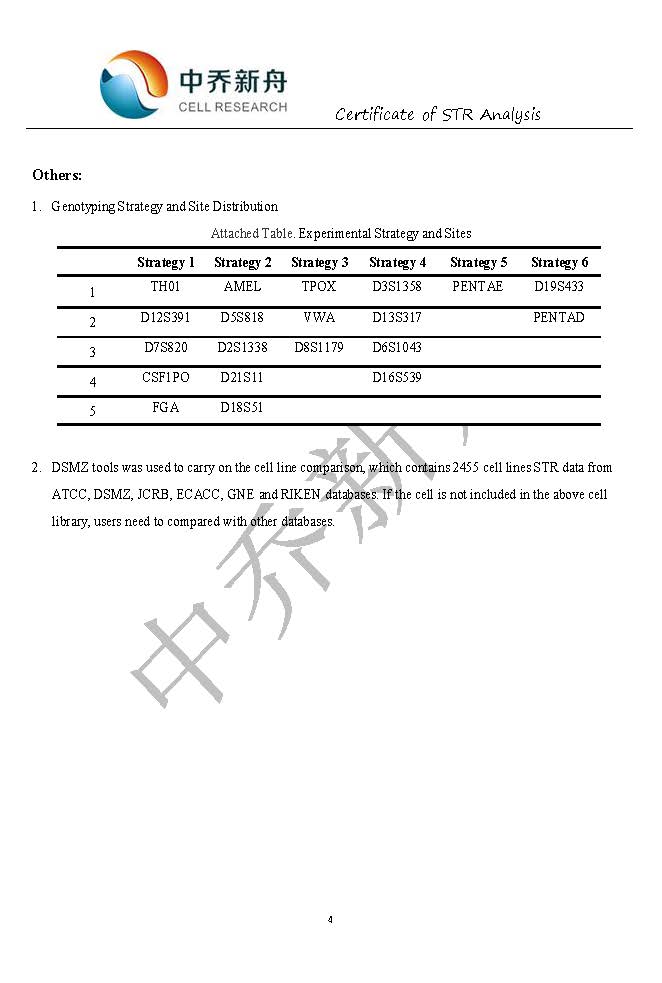

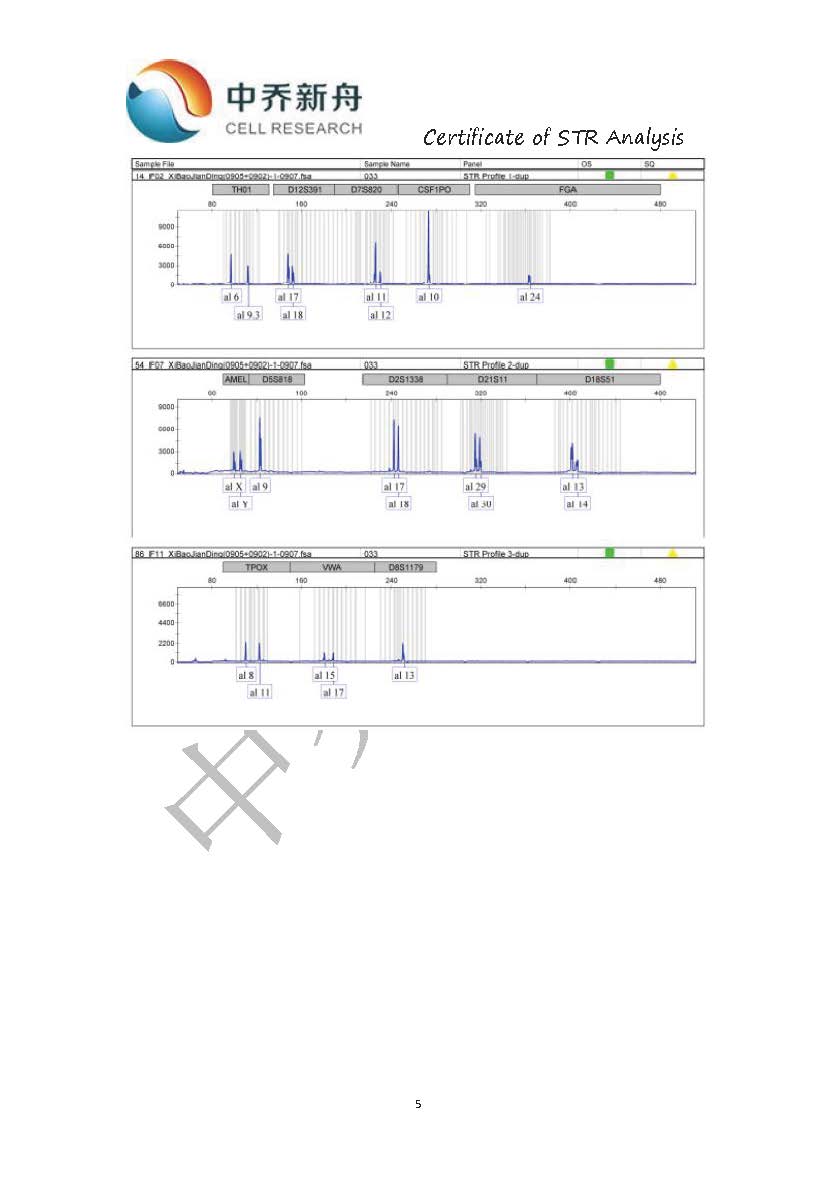

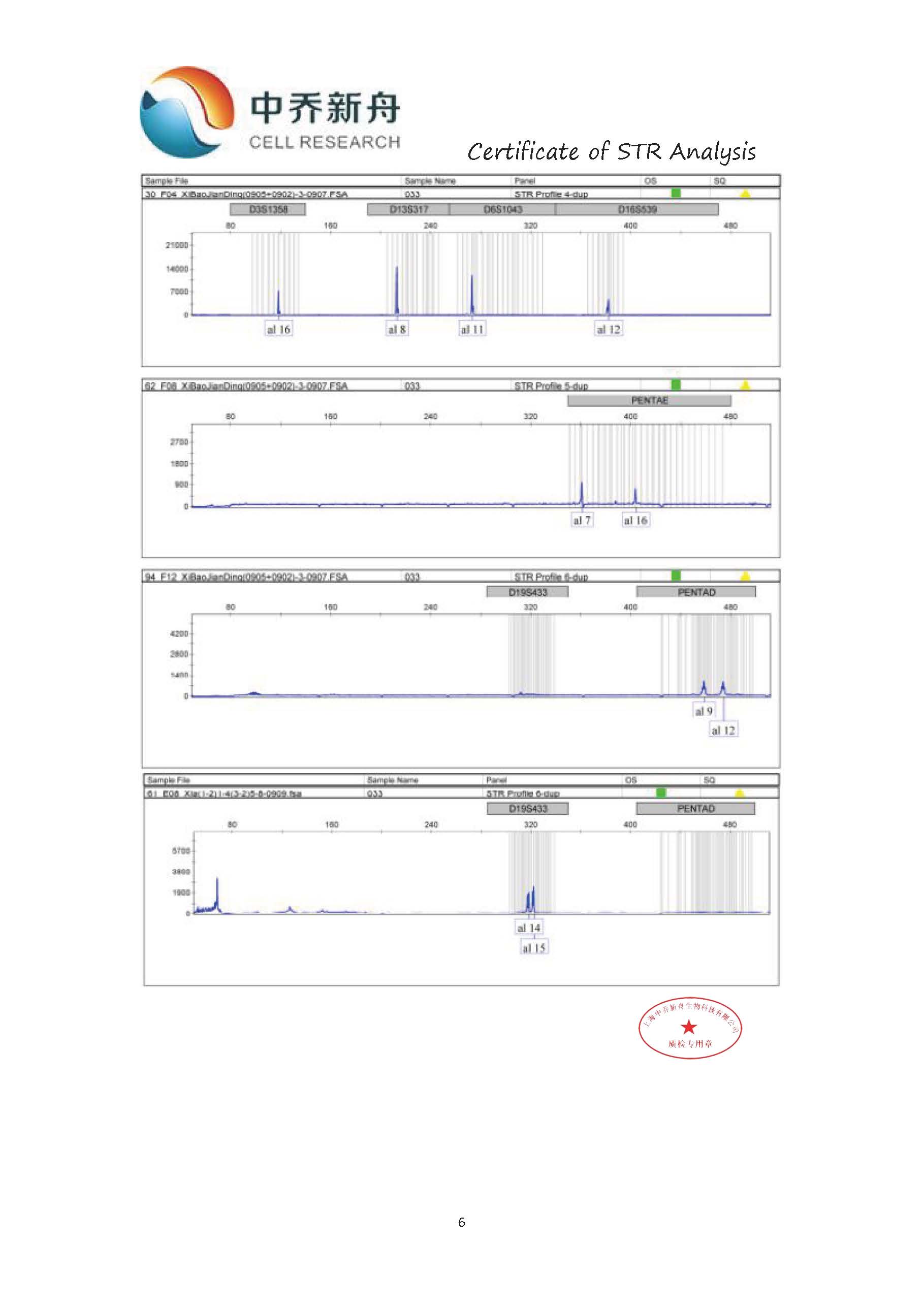


**Supplementary materials 3**
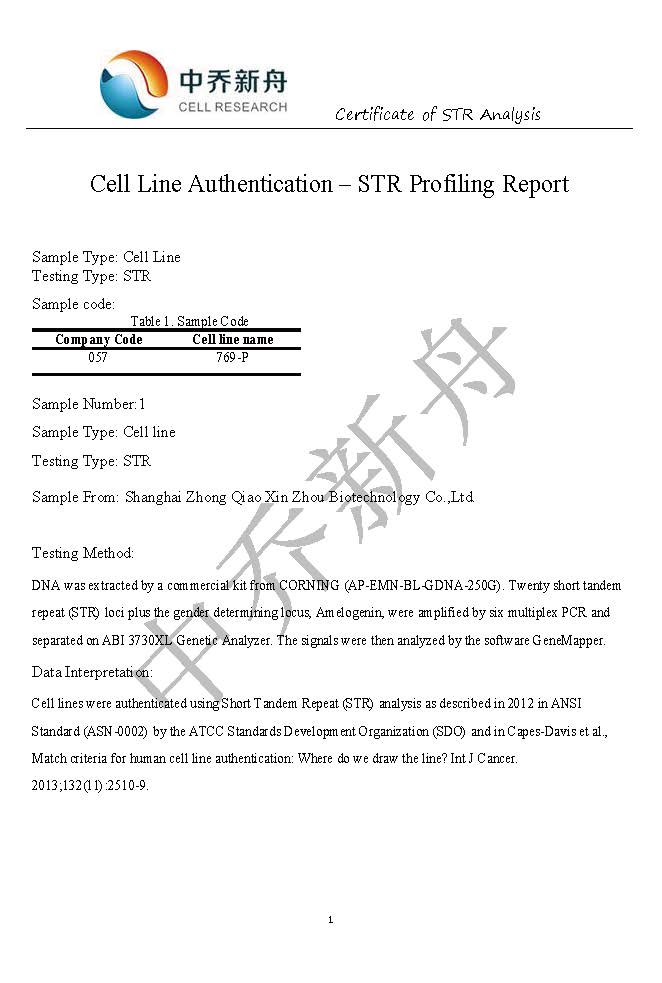


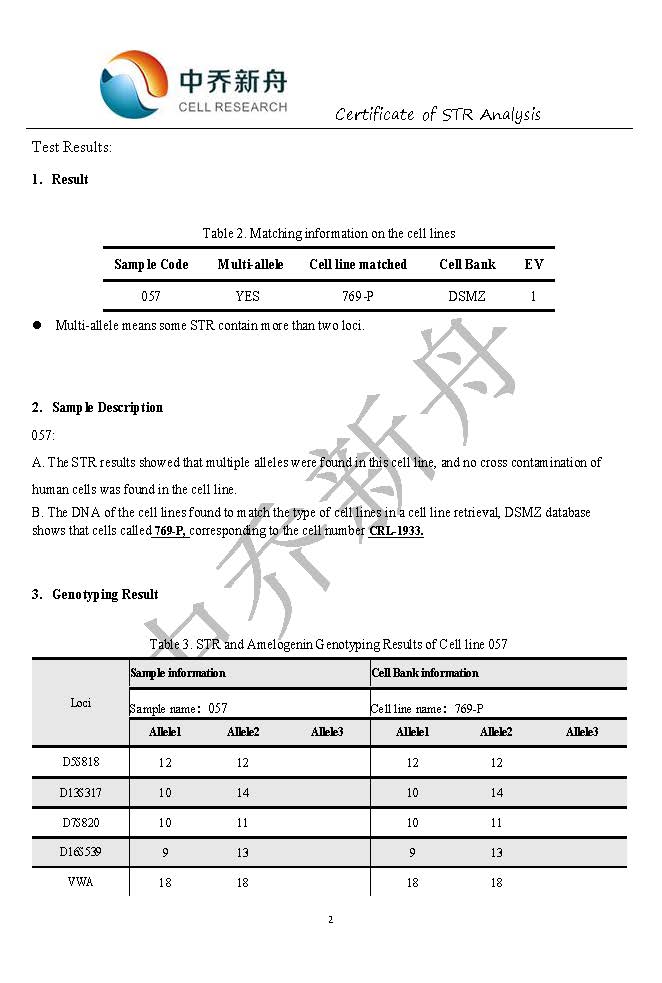

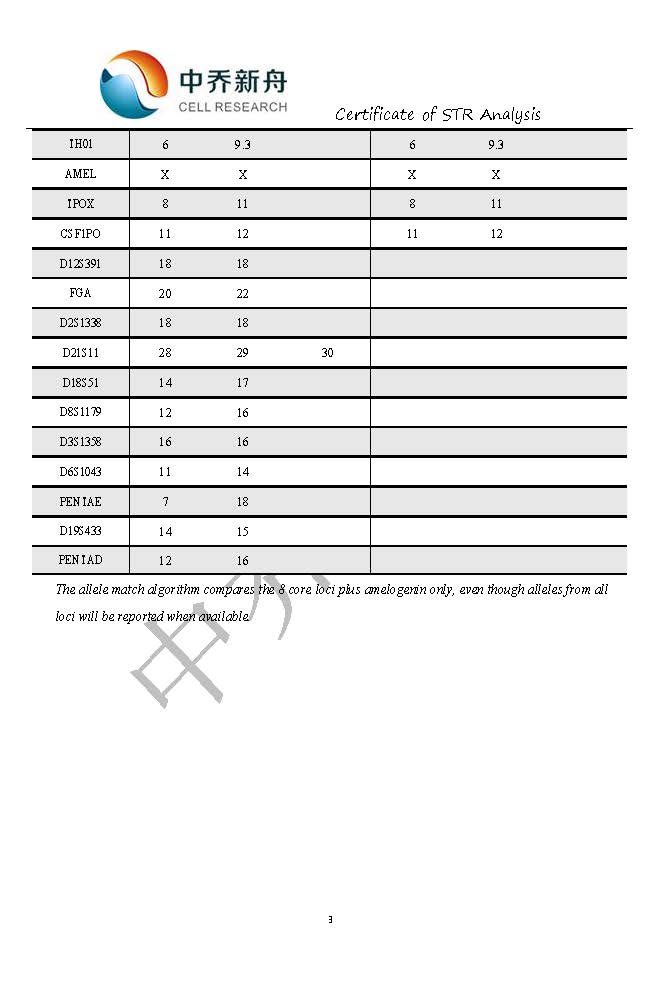

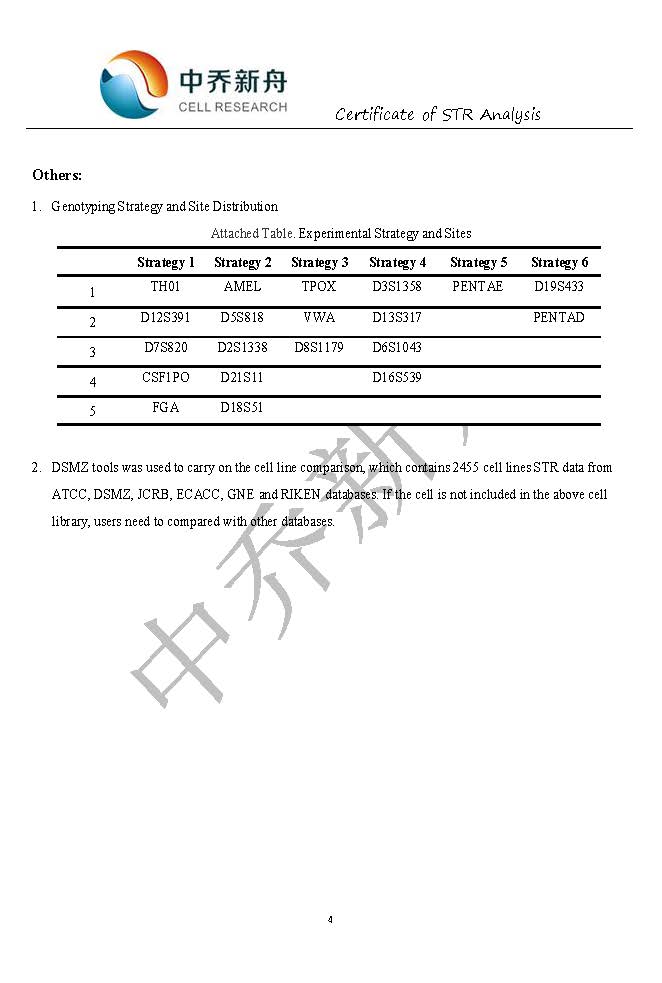

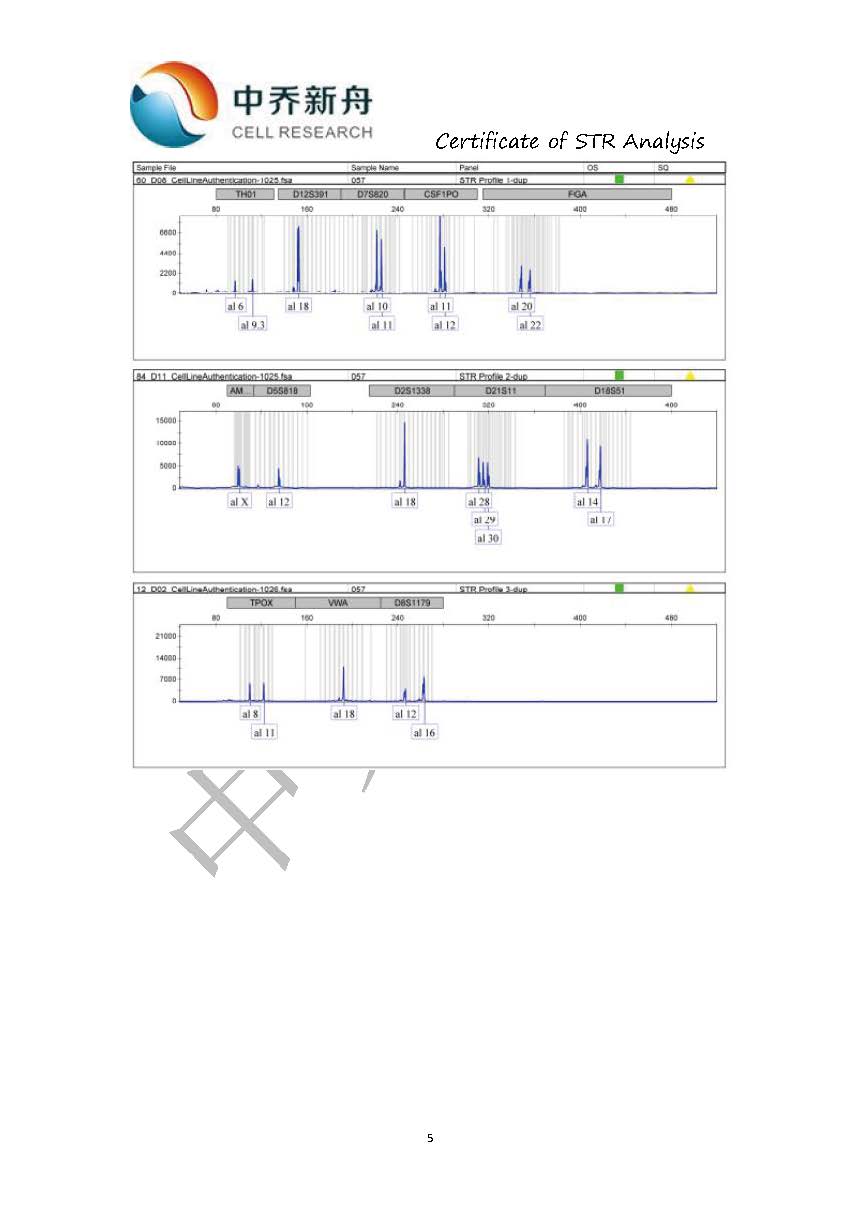

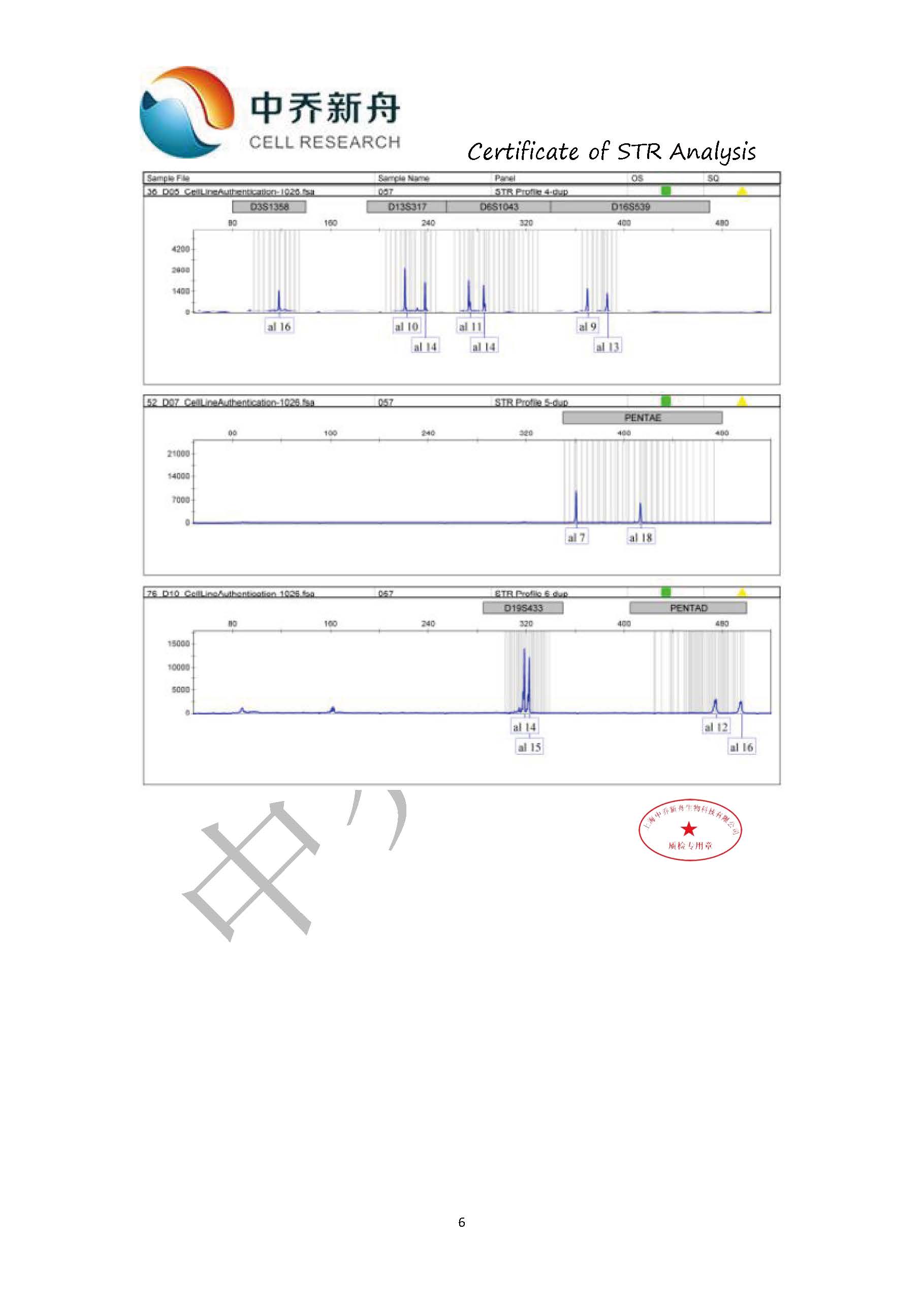

Supplement: Supplementary file 1 [file DataSheet1.docx]
